# Constructing Noise-Invariant Representations of Sound in the Auditory Pathway

**DOI:** 10.1371/journal.pbio.1001710

**Published:** 2013-11-12

**Authors:** Neil C. Rabinowitz, Ben D. B. Willmore, Andrew J. King, Jan W. H. Schnupp

**Affiliations:** 1 Department of Physiology, Anatomy and Genetics, University of Oxford, Oxford, United Kingdom; 2 Center for Neural Science, New York University, New York, New York, United States of America; Cold Spring Harbor Laboratory, United States of America

## Abstract

Identifying behaviorally relevant sounds in the presence of background noise is one of the most important and poorly understood challenges faced by the auditory system. An elegant solution to this problem would be for the auditory system to represent sounds in a noise-invariant fashion. Since a major effect of background noise is to alter the statistics of the sounds reaching the ear, noise-invariant representations could be promoted by neurons adapting to stimulus statistics. Here we investigated the extent of neuronal adaptation to the mean and contrast of auditory stimulation as one ascends the auditory pathway. We measured these forms of adaptation by presenting complex synthetic and natural sounds, recording neuronal responses in the inferior colliculus and primary fields of the auditory cortex of anaesthetized ferrets, and comparing these responses with a sophisticated model of the auditory nerve. We find that the strength of both forms of adaptation increases as one ascends the auditory pathway. To investigate whether this adaptation to stimulus statistics contributes to the construction of noise-invariant sound representations, we also presented complex, natural sounds embedded in stationary noise, and used a decoding approach to assess the noise tolerance of the neuronal population code. We find that the code for complex sounds in the periphery is affected more by the addition of noise than the cortical code. We also find that noise tolerance is correlated with adaptation to stimulus statistics, so that populations that show the strongest adaptation to stimulus statistics are also the most noise-tolerant. This suggests that the increase in adaptation to sound statistics from auditory nerve to midbrain to cortex is an important stage in the construction of noise-invariant sound representations in the higher auditory brain.

## Abstract 2

Along the auditory pathway from auditory nerve to midbrain to cortex, individual neurons adapt progressively to sound statistics, enabling the discernment of foreground sounds, such as speech, over background noise.

## Author Summary

We rarely hear sounds (such as someone talking) in isolation, but rather against a background of noise. When mixtures of sounds and background noise reach the ears, peripheral auditory neurons represent the whole sound mixture. Previous evidence suggests, however, that the higher auditory brain represents just the sounds of interest, and is less affected by the presence of background noise. The neural mechanisms underlying this transformation are poorly understood. Here, we investigate these mechanisms by studying the representation of sound by populations of neurons at three stages along the auditory pathway; we simulate the auditory nerve and record from neurons in the midbrain and primary auditory cortex of anesthetized ferrets. We find that the transformation from noise-sensitive representations of sound to noise-tolerant processing takes place gradually along the pathway from auditory nerve to midbrain to cortex. Our results suggest that this results from neurons adapting to the statistics of heard sounds.

## Introduction

Because our auditory world usually contains many competing sources, behaviorally important sounds are often obscured by background noise. To accurately recognize these sounds, the auditory brain must therefore represent them in a way that is robust to noise. Previous work has suggested that the auditory system does build such sound representations. In the auditory periphery, sounds are represented in terms of their physical structure, including any noise [1]–[3], while data from human imaging studies suggest that, in higher areas of auditory cortex (AC), relevant sounds are represented in a more context-independent, categorical manner [4]–[8]. However, we know very little about the neural computations that might generate noise invariance or where exactly along the auditory pathway this is achieved.

We do, on the other hand, know that the firing patterns of individual auditory neurons change with acoustic context. Numerous experiments have varied the statistics of sound stimulation, such as sounds' overall intensity, modulation depth, or contrast, or the presence of background noise. In response to these manipulations, auditory neurons from the periphery to primary cortex have been observed to change their gain [9]–[12], temporal receptive field shape (i.e., modulation transfer function, MTF) [9],[11],[13]–[17], spectral receptive field shape [18],[19], and output nonlinearities [20],[21], or they undergo more complex changes in response patterns [22],[23]. These changes have been explored or explained in terms of signal detection theory [11], efficient coding [17],[20],[24], or maintaining sensitivity to ecologically relevant stimuli [21],[23]. Such forms of adaptation—not to the repetition of a fixed stimulus, but to the statistics of ongoing stimulation—offer a plausible neural mechanism for the construction of noise-invariant representations. A population of neurons that adapts to the constant statistics of a background noise could become desensitized to that noise, while still accurately representing simultaneously presented, modulated foreground sounds.

Here, we investigated whether adaptation to stimulus statistics in the auditory system enables the brain to build noise-invariant representations of sounds. To do this, we carried out three experiments. First, we measured neural responses to complex sounds embedded in stationary noise, by recording from single units and small multi-unit clusters in the auditory midbrain and cortex and by simulating responses in the auditory periphery. We find that as one progresses through the auditory pathway, neural responses become progressively more independent of the level of background noise. Second, we measured how the coding of individual neurons in these auditory centers is affected by the changes in stimulus statistics induced by adding background noise. We find that there is a progressive increase through the auditory pathway in the strength of adaptation to the altered stimulus statistics. Third, we considered how the noise-dependent responses of individual units combine to produce population codes. Population representations are usually addressed only indirectly, for example, by summing up results from individual units (though see [25],[26]), but here we investigated these directly, by asking how well the original, “clean” sounds could be decoded from the population responses to noise-tainted stimuli. We find a progressive increase in the noise tolerance of population representations of sound. Moreover, neuron-level changes in the strength of adaptation and population-level changes in the noise tolerance of decoding are well correlated both within and across auditory centers. This suggests that adaptation to stimulus statistics may indeed be a neural mechanism that drives the construction of noise-tolerant representations of sound.

## Results

We recorded neural responses in the central nucleus of the inferior colliculus (IC) and the primary fields of the AC in ferrets, while presenting a set of natural sounds in high and low signal-to-noise ratio (SNR) conditions (referred to as “clean” and “noisy” below). We compared these recorded neural responses against a sophisticated model of sound representation in the auditory nerve (AN) [27]. The simulated auditory nerve (sAN) model captures the functional components of the auditory periphery from the middle ear to the AN, including the adaptation that occurs at synapses between inner hair cells and AN fibers.

**Figure 1:**
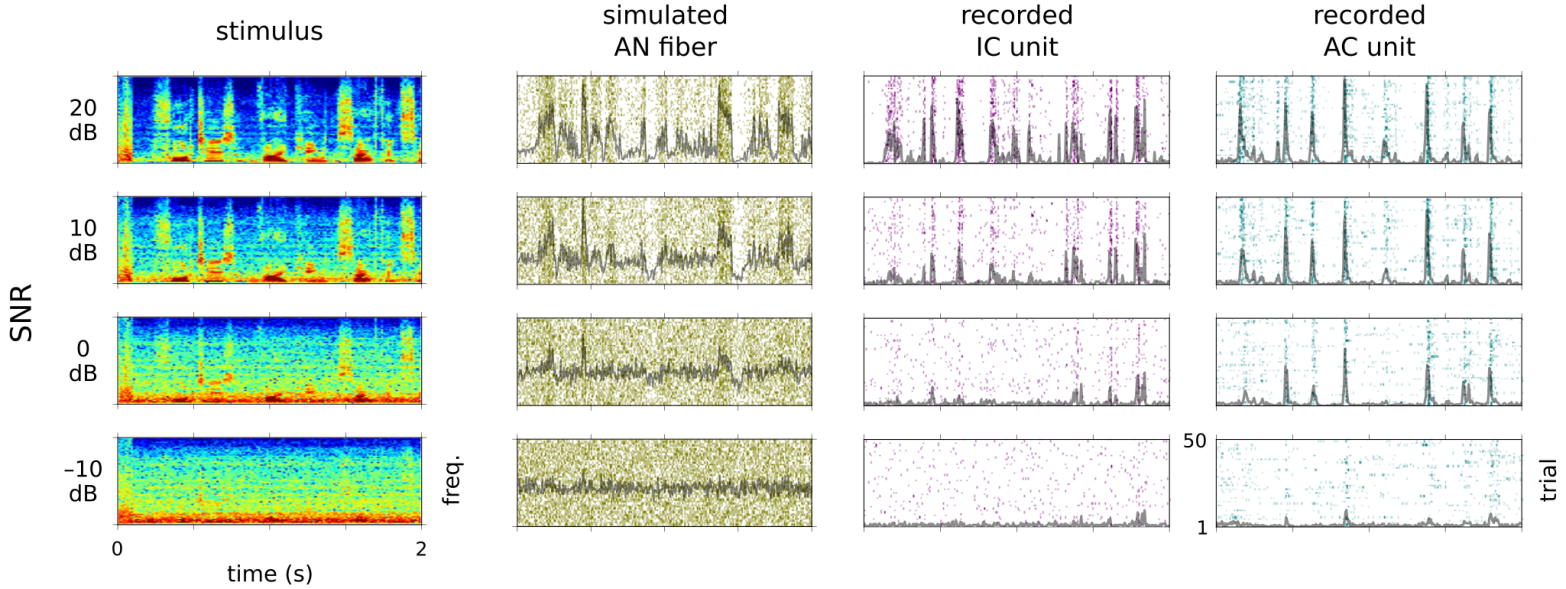
Single unit responses to clean and noisy sounds. Left column, the spectrogram of a segment of speech under four noise conditions, with the noise level increasing (i.e., the SNR decreasing) from top to bottom. Second to fourth columns, example rasters showing the responses of sAN responses and of responses recorded in the IC and AC, over 50 stimulus presentations. Gray lines, average PSTH.

We presented four audio segments (two speech, two environmental), to which spectrally matched noise had been added. In the “clean” condition, the SNR was 20 dB; in the “noisy” conditions, SNRs were 10 dB, 0 dB, or −10 dB (Figure 1). Fifty different noise tokens were used, so that responses reflected the average properties of the noise. We refer to the sounds in the clean condition as being the signal, and the sounds in the noisy conditions as being the signal plus noise. The noise we used was stationary—that is, its statistics did not change over time; it also had a flat modulation spectrum and no cross-band correlation. Such noises are exemplified by the sounds of rain, vacuum cleaners, jet engines, and radio static [17],[28]. We used this subclass of noise as such sounds are almost always ecologically irrelevant, and their statistics differ from those of relevant signals; the signal/noise distinction was therefore as unambiguous as possible. Very little sound signal was detectable to our ears in the noisiest condition, which lies close to the threshold of human and animal speech recognition abilities during active listening [25],[29]–[31].

**Figure 2:**
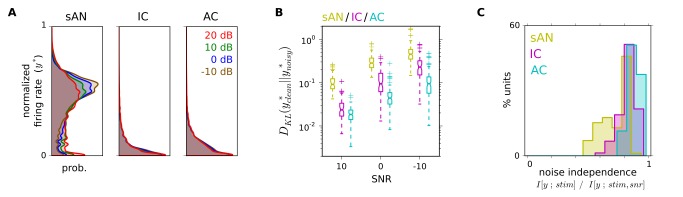
Along the auditory pathway, neurons' response distributions become increasingly independent of the level of background noise. (A) Average distribution of normalized firing rates by location/SNR. For each unit, 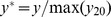, where 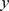 is the firing rate. This shows that the average response distribution within the population changes less with noise in higher auditory centers. (B) Kullback–Leibler divergence between individual units' normalized firing-rate distributions evoked from clean sounds and evoked from noisy sounds. Smaller values indicate that firing rate distributions were similar. This shows that individual neurons' response distributions change less with noise in higher auditory centers. (C) Statistical independence of stimulus-conditioned response distributions 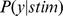 to the background noise level (see Materials and Methods for details of metric). Lower values indicate that response distributions were highly dependent on the stimulus SNR; a value of 1 indicates that response distributions were completely independent of the stimulus SNR. Median values of 0.80/0.84/0.88 for sAN/IC/AC (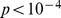, pairwise rank-sums tests).

For each auditory center (sAN, IC, AC), we measured how the neural coding of sounds changed as background noise was introduced. We found that, as we progressed from sAN to IC to AC, the distribution of neural responses became progressively more tolerant (i.e., less sensitive) to the level of background noise. This was evident at the gross level, as the distribution of sAN firing rates for each unit, 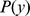, changed considerably as a function of the background noise level, while IC firing rates changed less, and AC even less so (Figure 2A–B). More notably, when we conditioned these response distributions on each 5 ms stimulus time bin, the response distributions 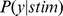 became more statistically independent of the background noise level from sAN to IC to AC (Figure 2C). This demonstrates that neural responses to complex sounds become less sensitive to background noise level as one ascends the auditory pathway.

### Adaptive Coding

**Figure 3:**
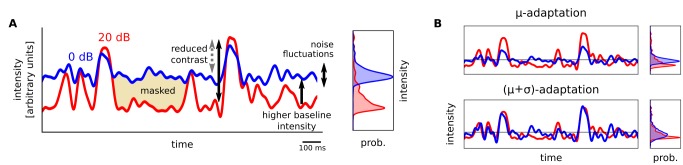
Effect of background noise on incoming signals within neurons' receptive fields. (A) Left, sound intensity within a cortical neuron's receptive field for clean (20 dB) and noisy (0 dB) stimulation (see Figure S1B). Right, distribution of the sounds' within-channel intensities. (B) Signals in (A) after adaptation to signal statistics.

What underlies this shift in coding, such that the responses of neurons in higher auditory centers are overall more tolerant to noise? To understand this, we considered three ways in which noise affects signals within auditory neurons' receptive fields (Figure 3A).

First, noise is an energy mask: when components of the original signal have intensities (within the receptive field) lower than that of the noise, they are obscured. Second, although the statistics of noise might not change over time, the noise itself is a time-varying stimulus, and auditory neurons may respond to noise transients [32],[33]. Because neurons in higher auditory centers progressively filter out faster temporal modulations [1], the energy of noise transients within neurons' linear receptive fields decreases from AN to IC to AC. However, simulations demonstrate that this alone cannot account for the observed differences in noise independence (Figure S1).

Finally, adding noise affects the statistics of the stimulus within the receptive field in two ways: it increases the baseline intensity, and it reduces the effective size of the peaks in intensity above the baseline—that is, it lowers the contrast. These effects can be roughly summarized as changing the mean (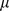) and standard deviation (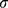) of the stimulus intensity distribution (which is, incidentally, non-Gaussian [24],[34],[35]).

If auditory neurons faithfully encoded stimuli within their receptive fields—irrespective of the stimulus statistics—then the response distributions would change their 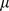 and 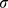 along with the stimulus distribution. However, if neurons adapted to the statistics—for example, by normalizing their responses relative to the local 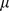 and 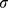
—then the response distributions would change less with the addition of noise (Figure 3B). Indeed, as shown above, the response distributions of sAN units changed considerably when noise was introduced, while those of IC units changed less, and cortex even less so. The increased noise tolerance in higher auditory centers may therefore result from a progressive increase in the strength of adaptation to stimulus statistics along the auditory pathway.

### 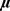
- and 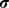-Adaptation Grow Stronger Along the Auditory Pathway

Given our reasoning above, we predicted that neuronal adaptation to 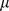 and 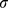 would increase along the auditory pathway. Previous experiments have shown that 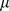-adaptation increases from AN to IC [20],[36] and that there is strong 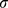-adaptation in AC [10],[12]; however, the overall changes in 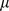
- and 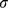-adaptation across the auditory pathway are unknown.

**Figure 4:**
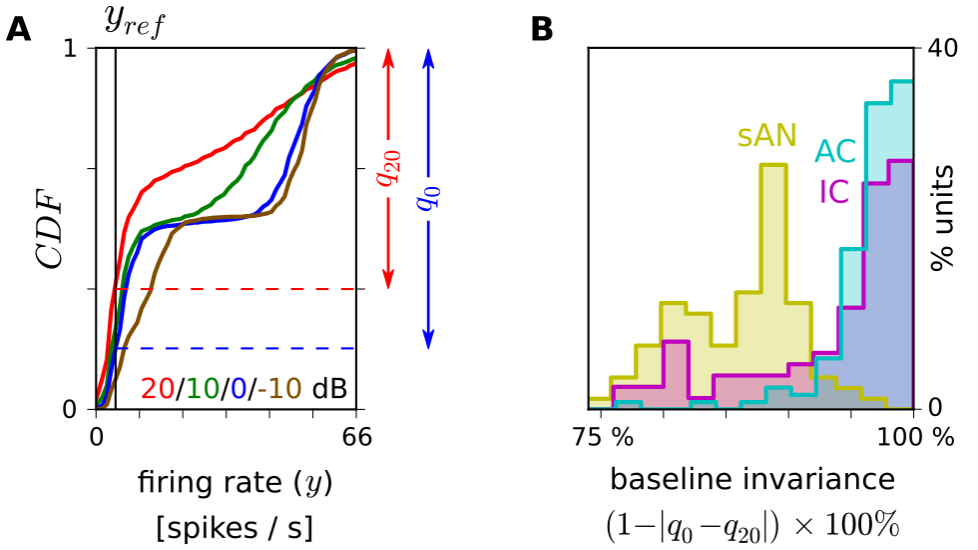
Increasing adaptation to stimulus baseline along the auditory pathway. (A) Calculation of BI, a measure of 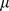-adaptation, for an example sAN fiber. CDF, cumulative distribution of firing rates. 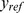, the 33rd percentile of the CDF under clean sound stimulation —that is, the firing rate with the cumulative probability 
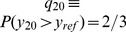
. BI indicates how little 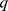 changes with SNR, as 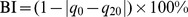. (B) Units' BI in each location.

We first tested the hypothesis that 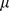-adaptation increases along the auditory pathway. Taking the neural responses to natural sounds, we quantified the degree to which introducing background noise changed the neural responses during the “baseline” periods of sound stimulation, such as when there was little stimulus energy within neurons' receptive fields to drive spiking. Rather than attempt to estimate neurons' receptive fields, we instead measured the relevant responses operationally. We defined a reference firing rate for each unit, 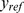, at the 33rd percentile of that unit's firing rate distribution during clean sound stimulation. We then calculated how often the firing rate exceeded 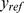 under different noise conditions (Figure 4A). The motivation for this measure is that, when 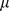-adaptation is weak, responses are sensitive to the baseline intensity of the stimulus, so adding noise should drive this value up. If 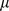-adaptation is strong, such that the neuron adapts out the increased baseline intensity of the stimulus, then the firing rate should exceed 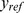 about as often in the noisy conditions as in the clean condition. We refer to these two possibilities as being of low, or high, baseline invariance (BI), respectively.

Introducing noise caused sAN fibers to change their firing relative to 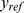 the most, and AC units the least (Figure 4B; median BI of 87/96/98% for sAN/IC/AC; 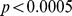). Similar results were obtained with 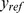 placed at other percentiles between 10% and 50%. This confirms that 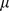-adaptation increases along the auditory pathway.

**Figure 5:**
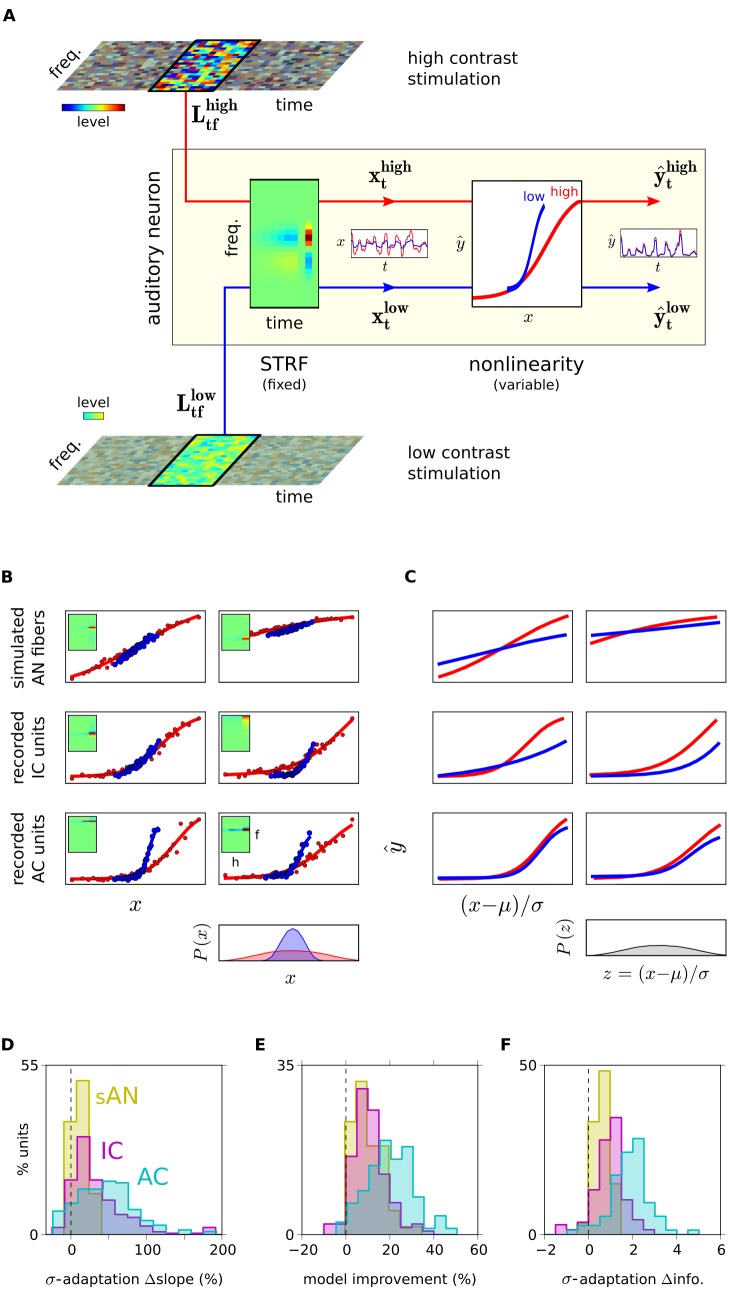
Increasing adaptation to stimulus contrast along the auditory pathway. (A) Schematic of adaptive-LN model. Top/bottom, DRC stimuli. DRCs are filtered through a STRF, then passed through an output nonlinearity, yielding the firing rate (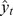). Output nonlinearities change with stimulus contrast. Insets, example time series. (B) Example units, nonlinearities during low (blue) and high (red) contrast DRCs. Insets, STRFs. Bottom, distributions of STRF-filtered DRCs under low/high contrast. (C) Nonlinearities in (B), replotted in normalized coordinates. (D) Contrast-dependent changes to the slope of units' nonlinearities. (E) Percentage of residual signal power explained by gain kernel model above an LN model [12]. (F) Log increase in Fisher information in units' encoding of low contrast stimuli, resulting from adaptation to this distribution. Zero, no adaptation. Larger positive values, greater adaptation.

We next tested the hypothesis that 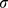-adaptation increases along the auditory pathway, by comparing how changes in contrast affect the gain of neurons at each location [10],[12]. We analyzed units' responses to dynamic random chord (DRC) sequences of differing contrasts (Figure 5A). DRCs comprise a sequence of chords, composed of tones whose levels are drawn from particular distributions. This allows efficient estimation of the spectrotemporal receptive fields (STRFs) of auditory neurons [37]–[39]. Varying the width of the level distributions allows parametric control over stimulus contrast. As in previous studies [10],[12], we modeled neuronal responses using the linear–nonlinear (LN) framework [40],[41], assuming that each neuron had a fixed (i.e., contrast-independent) STRF and a variable (contrast-sensitive) output nonlinearity. Contrast-dependent changes in coding are thus revealed through changes to output nonlinearities [10],[12].

Changing contrast had little effect on sAN coding, but caused small gain changes for IC units, and large gain changes for cortical units (Figure 5B; further examples in Figure S2). Higher in the auditory pathway, contrast-dependent gain changes were stronger (sAN/IC/AC medians: 11/27/44%; 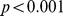; Figure 5D), occurred on slower timescales (time constants 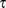 negligible/35/117 ms for sAN/IC/AC; 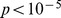; Figure S3), and were more important to adaptive-LN model predictive power (median improvement over LN model for sAN/IC/AC: 8/10/20%; not significant for sAN vs. IC, 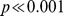 otherwise; Figure 5E) [12]. We confirmed this with a Fisher information analysis: by comparing how much Fisher information a unit typically carried in its firing rate about a low contrast stimulus when it was adapted to low contrast with the amount it typically carried about the same stimulus when it was adapted to high contrast, we found that contrast-adaptive changes in coding were more profound higher up in the auditory pathway (Figure 5F; median 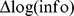 of 0.6/1.0/2.0 for sAN/IC/AC; 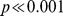). Thus there is an increase in 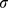-adaptation along the auditory pathway.

### Population Representations of Sound

Given that 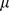
- and 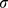-adaptation increase along the auditory pathway, how does this affect the representation of complex sounds by populations of auditory neurons? To answer this, we used a stimulus reconstruction method [42]–[45] that quantified how accurately the spectrogram of a presented sound could be reconstructed from the neuronal responses of each population.

**Figure 6:**
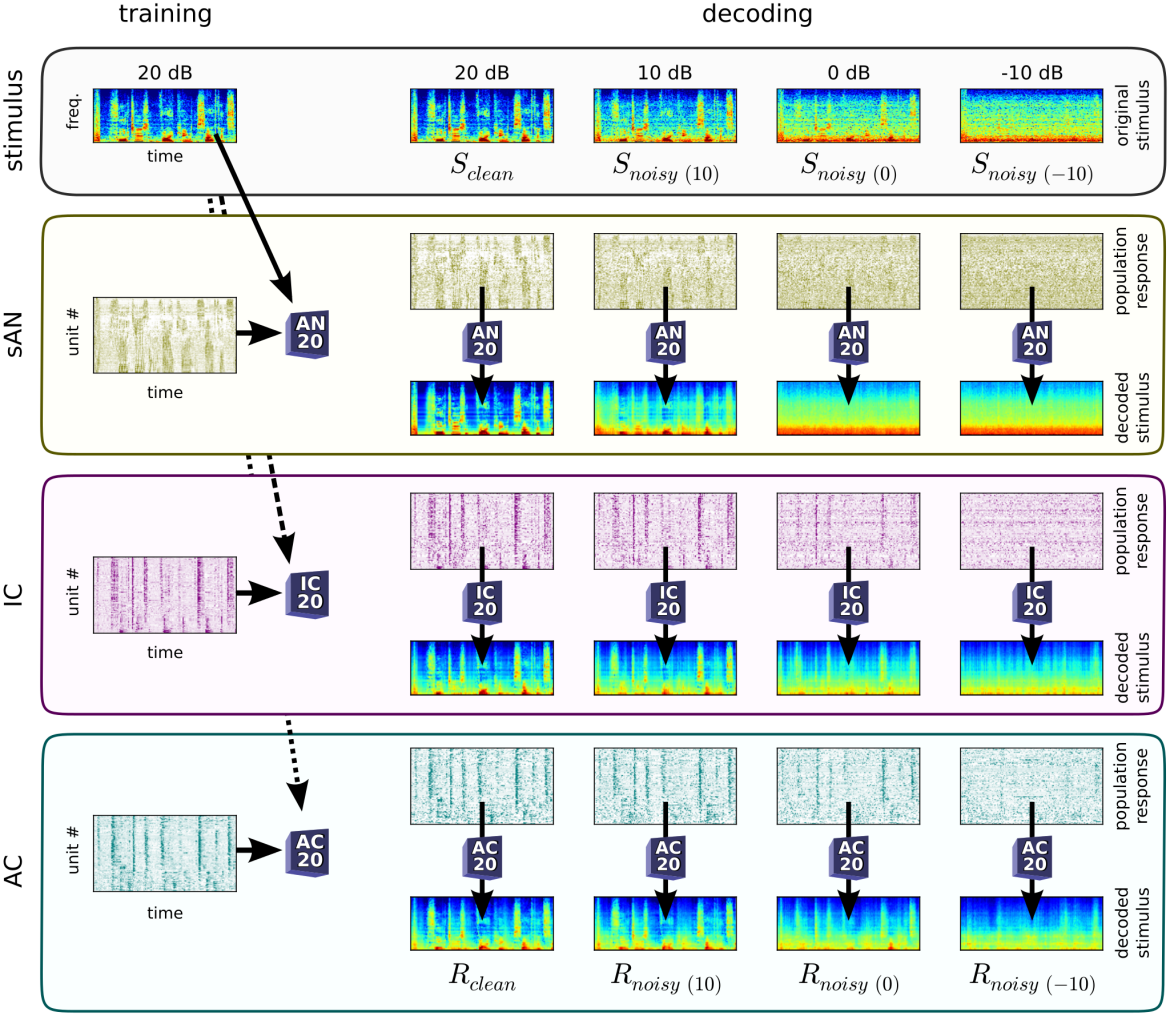
Decoding the population representations of clean and noisy sounds. Schematic of the decoding of neural responses. For each auditory center, a decoder was trained to reconstruct the clean sound spectrogram from the population responses to the clean sounds. We then measured the performance of these decoders when reconstructing spectrograms from the responses to both clean and noisy sounds. Top row, spectrogram of a 2(20 dB SNR) and noisy (10/0/−10 dB SNR) conditions. Left column, decoder training from responses to clean sounds. Population responses are shown as neurograms: each row depicts the time-varying firing rate of a single unit in the population; rows are organized by CF. Right, reconstructed spectrograms (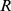) from population responses to noisy sounds, using the same decoders as trained on the left. The similarity between the reconstructed spectrogram 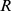 and the presented spectrogram 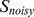 is measured by 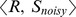; likewise, the similarity between 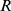 and the original, clean spectrogram 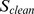 is measured by 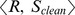. The tendencies for the sAN decoder to produce 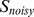-like spectrograms, and the IC and AC decoders to produce 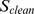-like spectrograms, are most visible for the 0 dB and −10 dB conditions.

The reconstruction was done as follows. We first trained a spectrogram decoder on the population's responses to clean sounds (Figure 6). This decoder was based on a dictionary approach (see Materials and Methods section “Population Decoding”). We then tested the decoder on a novel set of responses to clean sounds and measured how close the reconstructed spectrograms, 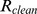, were to the original sound spectrograms, 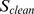, using a similarity metric, 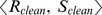. These measurements quantify the degree to which the spectrogram of the clean sounds was encoded in the population responses.

**Figure 7:**
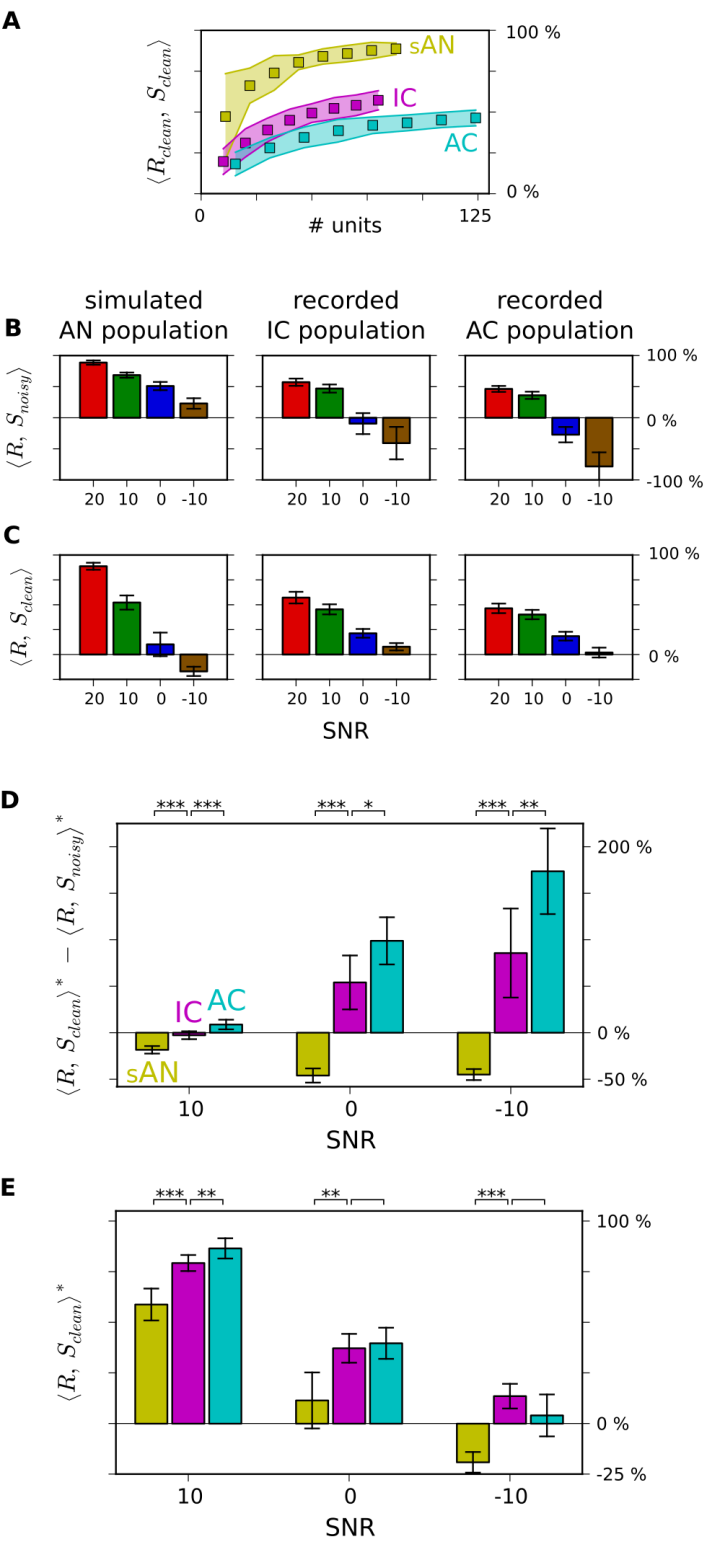
Population representations of natural sounds become more noise-tolerant along the auditory pathway. (A) Similarity between decoded responses to the clean sounds (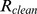), and the clean sounds' spectrograms (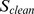). Abscissa, sampled population size. Colored areas, bootstrapped 95% confidence intervals. (B–C) Similarity between decoded responses to the noisy sounds (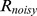), and the spectrograms of the presented, noisy sounds (B), or the spectrograms of the original, clean sounds (C). Reconstructions are from the full populations in each location. Red bars are the same in (B) and (C), denoting 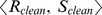 (i.e., the rightmost points for each curve in A). Error bars, bootstrapped 95% confidence intervals. (D) Index of whether decoded responses were more similar to the presented, noisy sound (negative values), or the original, clean sound (positive values). Similarities denoted by asterisks (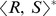) are normalized to the maximum score for each location, 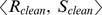. Error bars, 95% confidence intervals. Pairwise comparison statistics (bootstrapped): 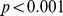 (***), 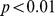 (**), 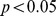 (*). (E) Decoder accuracy in recovering the clean sound's identity from noisy responses, relative to accuracy in doing so from clean responses.

For all three auditory centers, reconstruction accuracy increased with population size (Figure 7A). The best reconstructions were available from sAN responses; reconstructions from IC and AC were less accurate. This is likely to be due to several factors. In particular, the synthetic sAN population provided more uniform coverage of the frequency spectrum (Figure S4), and contained less trial-to-trial variability than the recorded data. Also, both IC and AC are well known to have greater low-pass modulation filtering [1], which should reduce the overall fidelity of the spectrogram encoding at these higher auditory centers.

### What Is Being Encoded by Neural Populations?

Our interest was not in the absolute performance of these decoders, but rather in how the stimulus representations changed with the addition of background noise. We began by asking, what are sAN, IC, and AC encoding in their population responses? This is a difficult question to address since the dimensionality of a population response is very high. We therefore recast this problem as follows. We considered a scenario where the higher brain has learned to recognize sounds in the absence of noise, based on the respective encodings in sAN, IC, and AC. We then asked what would happen if the brain then tries to extract sound features from responses to the noisy sounds, if it is assumed that neural populations encode sound features in exactly the same way as when noise was absent.

We considered two hypotheses for what might happen. First, when the brain attempts to reconstruct stimulus features from the noisy sounds, it might accurately recover the whole sound mixture, containing the superimposed signal and noise. Alternatively, the reconstructed stimulus might include the signal alone, and not the noise. We denote these two possibilities as “mixture”-like and “signal only”–like representations. These are two ends of a spectrum: the sAN, IC, and AC populations may show different degrees of “mixture”-like and “signal only”–like coding.

To test these hypotheses, we used the same decoders (which had already been trained on the clean stimuli) to reconstruct the stimulus spectrograms from the responses of the three populations to the noisy sounds. We quantified how the accuracy of the reconstructed spectrograms (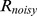) changed across noise levels, by measuring the similarity of 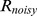 both to the presented, noisy spectrograms (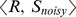; Figure 7B) and to the spectrogram of the original, clean sound (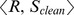; Figure 7C). To be able to compare these values across different populations, we normalized these measurements, by dividing them by that population's value of 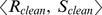 (the absolute performance of the decoder on the clean sound responses). We denote the normalized values as 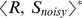 and 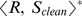, respectively.

The rationale for these measurements was as follows. If the reconstructed spectrogram contains both the signal and the noise, then 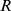 should be more similar to the spectrogram of the noisy, presented sound, 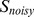, than it is to the spectrogram of the original, clean sound, 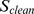, which contains the signal alone. Thus, 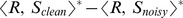 would be less than 0. On the other hand, if the reconstructed spectrogram contains the signal, but not the noise, then 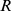 should be more similar to 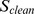 than to 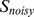, and so 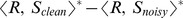 would be greater than 0.

For the sAN responses, we found that 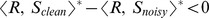. This indicates that, using a fixed decoder, both the signal and the noise are extracted from the sAN responses. In other words, the noise directly impinges on the encoding of the signal in the sAN responses. The reverse was true for AC, where 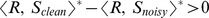. This indicates that, using a fixed decoder, the signal can be extracted from the AC responses, without recovering much of the noise. The IC responses lay between these two extrema (Figure 7D).

It is important to emphasize here that this does not imply that noise features are altogether discarded by the level of the cortex, and not represented at all. The decoders here were specifically trained to extract the clean signal; these results therefore highlight how much or how little the encoding of the original signal is affected by the addition of background noise. As we used new noise tokens on each presentation, it was not possible to train decoders to extract the noise in the mixture from the response (rather than the clean sound), nor to accurately determine the extent to which transient noise features can be recovered from population responses. We therefore treat the noise here as a nuisance variable—that is, as a distractor from the encoding of the ecologically more relevant components of the sound signal.

In sum, while population representations in the periphery are more “mixture”-like, insofar as stationary noises are encoded in a similar way as complex sounds, there is a shift towards more “signal only”–like population representations in midbrain and then cortex, wherein stationary noise is not encoded together with the foreground sound.

### Noise-Tolerant Population Representations of Sound

We next asked a related but different question: If we start with a population representation of the clean sound, how tolerant is this representation to the addition of background noise? Unlike the question above, this requires us to take into account that the addition of noise degrades any reconstruction (Figure 7B–C).

To measure noise tolerance, we reasoned as follows. The decoder estimates a relationship between the population response and the clean sound spectrogram (i.e., the signal). If a population representation is noise-tolerant, such that the response does not change considerably when background noise is added, then 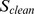 should be as accurately recovered from responses to the noisy sounds as it is from the clean sounds (i.e., 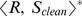 should be high). Conversely, if the population representation is noise-intolerant, such that the response changes considerably when background noise is added, then 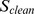 should be more poorly recovered from responses to the noisy sounds than from responses to the clean sounds (i.e., 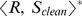 should be low). We found that for moderate noise levels, the value of 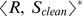 was highest for the AC, and lowest for the sAN (Figure 7E). This suggests that cortex maintains a more consistent representation of the signal as noise is added.

Thus, the population representations of sound change through the auditory pathway. In the periphery, neural populations that encode the signal also encode the noise in a similar way, responding to features of the mixed input. By the level of the cortex, however, neural populations represent the signal in a more noise-tolerant fashion, by responding to the sound features that are common between clean and noisy conditions.

### Adaptive Coding Partially Accounts for Noise-Tolerant Populations

Earlier, we demonstrated that adaptation to stimulus statistics increases along the auditory pathway. We therefore asked whether this could account for how background noise affects population representations of complex sounds along the auditory pathway.

To develop this hypothesis, we simulated populations of model auditory neurons with variable degrees of adaptation to sound statistics (Figure S5). These simulations confirmed that increasing 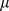-adaptation and 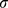-adaptation could account for the decoder results shown in Figure 7D–E. In particular, the simulations made two specific predictions. The first is that the increase in 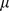-adaptation along the auditory pathway may be responsible for the shift from encoding 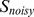 (in sAN) to 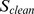 (in AC), as observed in Figure 7D. This is because 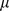-adaptation would remove the strong differences in response baselines between the representations of clean and noisy sounds (Figure 3B, top). The second prediction is that the increase in 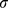-adaptation along the auditory pathway could be responsible for the increased tolerance of 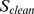 decoding to the addition of noise, as observed in Figure 7E. This is because 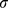-adaptation rescales the representation of the stimulus, such that the peaks in intensity are relatively independent of the noise level (Figure 3B, bottom).

**Figure 8:**
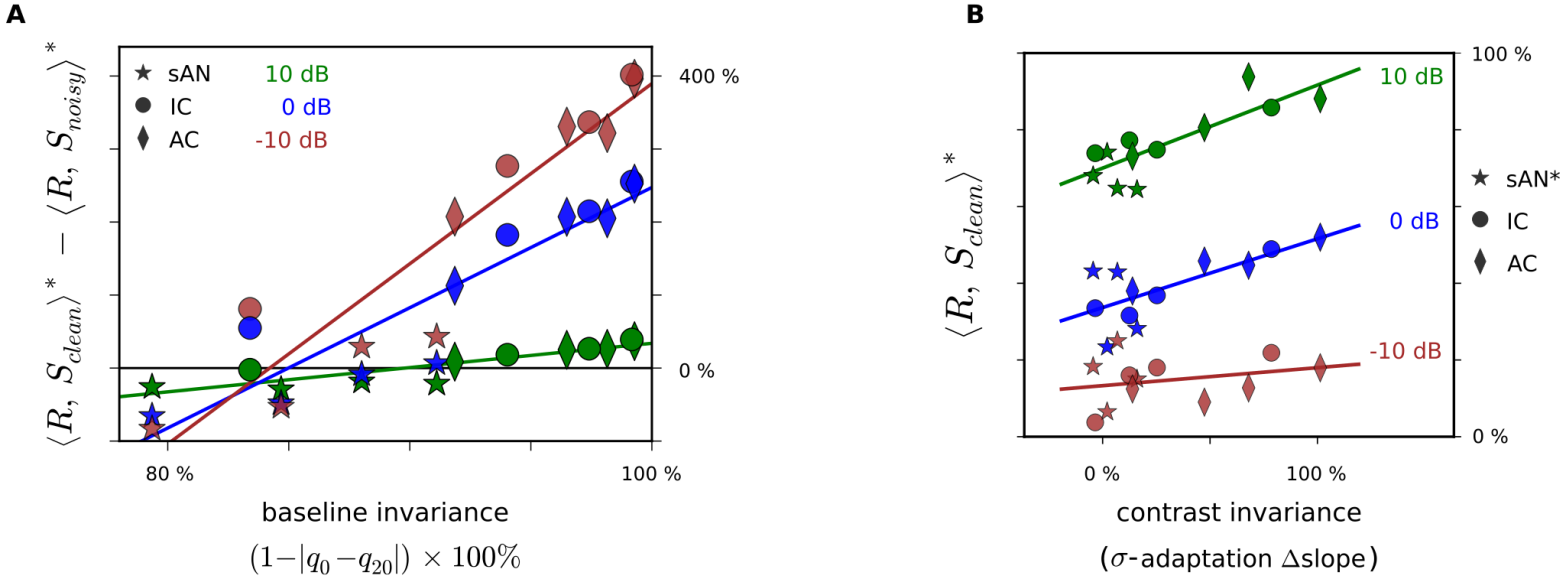
Higher 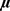
- and 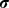-adaptation explain the increased noise-tolerance of population representations. (A) Relationship between decoder performance and BI (measure of 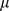-adaptation). Each point represents a subpopulation (one quarter) of the units from each of the sAN/IC/AC populations, subdivided according to units' BI (values in Figure 4B). Abscissa, mean BI in the subpopulation. Ordinate, performance of the subpopulation decoder. Lines, linear fit per SNR. (B) Relationship between decoder performance and CI (measure of 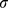-adaptation), similar to (A). Here, each point represents a subpopulation (one quarter) of the units from each of the sAN/IC/AC populations, subdivided according to the amount of units' contrast adaptation (values in Figure 5D). sAN values of 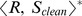 were adjusted for low BI (see Figure S6).

To test the first prediction—that 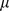-adaptation drives populations to represent 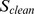 rather than 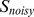
—we subdivided each neuronal population into four groups according to the neurons' baseline invariance (BI; our measure of 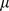-adaptation). For example, in IC, the 20 neurons with lowest BI formed a subpopulation with mean BI of 83%, and the 20 neurons with highest BI formed a subpopulation with mean BI of 99%. We then decoded responses from each of the 12 subpopulations. We found that the subpopulations with larger BI yielded more 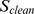-like spectrograms upon decoding (Figure 8A). That is, neurons with stronger adaptation to baseline sound intensity showed more “signal only”–like coding than “mixture”-like coding. This factor largely explained the differences in 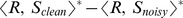 between each level of the pathway (Table S1A).

To test the second prediction—that 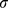-adaptation drives populations to encode 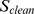 in a more noise-tolerant fashion—we again subdivided each population into four groups, by sorting units by their contrast-dependent gain changes—that is, the extent of their contrast invariance (our measure of 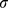-adaptation). Those subpopulations with stronger contrast-dependent gain control yielded 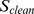-representations that degraded less with the addition of noise. This factor largely explained the differences in 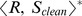 across auditory centers (Figure 8B, Table S1B). Together, these results support the notion that adaptation to stimulus statistics is an important mechanism that drives populations of auditory neurons to represent sounds a noise-tolerant way.

## Discussion

Our data show that, as one progresses along the auditory pathway from the AN to IC to AC, neurons show increasing adaptation to the mean (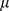, Figure 4) and contrast (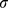, Figure 5) of sounds. This adaptation to stimulus statistics is relevant to hearing in noisy environments, because an important effect of background noise is to change these sound statistics. By adapting to such changes, populations of neurons could, in principle, produce a relatively noise-invariant code for nonstationary sounds (Figure 3). Consistent with this hypothesis, we found that population representations of natural sounds in higher auditory centers show stronger tolerance to the addition of stationary background noise (Figure 7), and that this noise tolerance could largely be explained by increases in 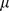
- and 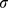-adaptation (Figure 8). This suggests that the increase in adaptation to stimulus statistics along the auditory pathway makes an important contribution to the construction of noise-invariant representations of sound.

### Towards Normalized Representations

The effect of 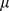
- and 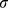-adaptation can be understood by representing the structure of a sound as a time-varying function, 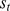. The brain does not have direct access to 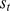; instead, when the sound is produced at a particular amplitude (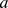) and is heard against a background of other sounds (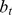), the signal that the ear actually receives is the sound mixture 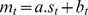. To identify a sound, the brain must recover the sound structure, 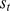, without being confused by the often irrelevant variables 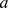 and 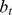.

Experiments with synthetic DRC stimuli show a shift in coding away from a raw signal (resembling 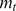) in the periphery toward a more normalized signal (resembling 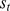) in the cortex. When the contrast of DRCs is manipulated, we find that sAN responses to DRCs are reasonably well described by an LN model without gain changes. Their firing rate is a function of 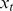
—that is, the DRC filtered through that neuron's STRF (Figure 5B). This suggests that the AN, as a whole, provides a relatively veridical representation of sound mixtures reaching the ear. In comparison, many cortical units, and some IC units, adapt to changes in DRC contrast by changing their gain. These units' firing rates are not a function of 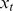 (as in the sAN); they are often better described as a function of a normalized variable, 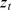, in which the stimulus contrast (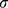) has been divided out (Figure 5C). Even though AC neurons do not show complete contrast-invariance for these stimuli (the median AC gain change was 44%; perfect 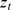-encoding would be 100% gain change; Figure 5D), AC neurons' responses depend less on stimulus contrast than those in IC or sAN. A similar shift in coding is evident when considering small changes in the mean level of a DRC. Whereas each sAN fiber provides a relatively fixed representation of 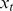, IC and AC units adjust their baseline firing rates so that they effectively subtract out the stimulus mean (Figures S7 and S8). The effect of adaptation to stimulus statistics is thus that cortex (and, to a lesser degree, IC) provides a sound representation that is closer to the underlying sound, 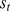, than to the sound mixture reaching the ear, 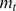.

### Functional Mechanisms for Building Noise-Invariant Representations

It is likely that adaptation to stimulus statistics is one of several changes in neural coding that contributes towards the construction of noise-invariant representations of sounds. Related findings were obtained by Lesica and Grothe [17], who studied changes in MTFs of IC neurons under noisy stimulation. Just as our investigation of 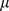
- and 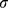-adaptations was initially motivated by considering how the statistics of within-receptive field signals would change under clean and noisy sound stimulation (Figure 3), so Lesica and Grothe began by investigating the difference in the amplitude modulation spectra between foreground vocalizations and background noises. They observed that vocalizations contain more power in slow (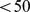
Hz) amplitude modulations than background noises. When the authors presented vocalizations to gerbils and recorded from neurons in the IC, they found that single units' MTFs shifted from being bandpass to more lowpass, suggesting that IC neurons redirect their coding capacity to modulation bands of higher SNR under noisy conditions.

Similar results were recently obtained by Ding and Simon [8], who measured the aggregate activity in human AC via magnetoencephalography, as subjects listened to speech in spectrally matched noise. They found that as background noise is added to speech, the entrainment of aggregate cortical activity to slow temporal modulations (<4 Hz) in the speech signal remains high, while entrainment to faster (4–8 Hz) modulations degrades with noise. Since the gross envelope of the original speech can be decoded from aggregate responses to the clean and noisy stimuli, noise induces a change in response gain as well as changes to MTFs.

The relationship between our observations of increasing 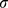-adaptation from periphery to cortex, and these previous findings of changing MTFs in IC neurons and aggregate cortical activity, may depend on the modulation specificity of the gain changes. For instance, a nonspecific increase in neural response gain would manifest as an overall upwards shift in the MTF. Conversely, an upwards shift within a small region of the MTF corresponds to a modulation-band–specific increase in gain. One possibility is that during complex sound stimulation, auditory neurons determine their gain independently for different modulation “channels” (such as described in modulation filterbank models [28],[46]), as a function of the signal statistics within each channel. This might have different effects on MTFs depending on the modulation spectrum of the background noise. In indirect support of this possibility, the extent to which the coding of different cells is affected by a given background noise appears to depend on each cell's modulation tuning [47]. An alternative possibility is that auditory neurons might always become more modulation lowpass in the presence of background noise, regardless of the noise's actual modulation statistics. This might reflect a set of priors about what is signal and what is noise in an incoming sound mixture. Our set of unique sounds and background noises was too small to test these two hypotheses (or even to measure MTFs). Nevertheless, if auditory neurons additionally demonstrate modulation-specific gain in response to noise, it is likely that this effect grows stronger from periphery to cortex.

These data also provide some insight as to how our results might extend to more complex classes of background noise. Here, we have characterized coding changes induced by adding stationary noise with flat modulation spectra and no cross-band correlations. Many background sounds have more complex (often 1/*f*-like) modulation spectra [28],[35]; a greater proportion of their modulation energy lies within the common passband of midbrain and cortical auditory neurons. Since our simulations suggest that greater modulation tuning plays only a small part in enabling tolerance to noise with flat modulation spectra, it should be less important still for enabling tolerance to noise with 1/*f*-like modulation spectra. We therefore expect that the adaptive coding we and others describe is crucial for more general classes of background noise. Beyond this, some background sound textures also contain correlations across carrier or modulation channels [28], while others are nonstationary, changing their statistics over time. An understanding of how these noise features differentially affect signal encodings along the auditory pathway would require further experiments utilizing a broader set of background noises.

An alternative hypothesis for how the brain builds noise-invariant representations of sound is that the very nature of these representations may be changing along the auditory pathway, from an emphasis on encoding predominantly spectrotemporal information in the periphery to encoding information about the presence of higher level auditory features in cortex. This, for instance, is a position recently argued for by Chechik and Nelken [48], based on their investigation of the responses of cat cortical neurons to the components of natural birdsong. Emerging data from the avian brain support this idea: the avian analogue of AC appears to shift its encoding toward sparse representations of song elements, which can be encoded in a noise-robust manner [49]. Our results relate to this hypothesis by emphasizing that, to the extent that the mammalian midbrain and cortex do encode spectrotemporal information about ongoing sounds, they do so in progressively more normalized coordinates. This captures at least some (but likely not all) of the proposed representational shifts from periphery to cortex.

Finally, bottom-up mechanisms are undoubtedly just a part of a broader infrastructure for selecting and enhancing representations of particular sounds heard within complex acoustic scenes. In our experiments, we chose stimuli for which the assignment of the tags “signal” and “noise” (or “foreground”/“background,” or “relevant”/“irrelevant”) to components of the mixture is reasonably justified by the different statistical structures of natural and background sounds [17],[28],[35],[50]. On the other hand, there are also many real-world situations for which such assignment is ambiguous, and depends on task-specific demands. Listening to a single talker against a background of many is one notable instance. Yet human imaging studies reveal that in such circumstances, the neural representation of attended talkers is selectively enhanced relative to that of unattended talkers, even at low SNRs [7],[26],[51]. While noise tolerance appears to grow even stronger between core and belt AC [7],[8], this is likely to be attention-dependent [7],[8],[52]–[54]. Understanding how we create noise-tolerant representations of sound within more complex mixtures is thus interwoven with questions of how we segment these scenes, how we tag the components as “signal” and “noise,” and how we direct our attention accordingly.

In sum, our results provide a clear picture of a bottom-up process that contributes to the emergence of noise-invariant representations of natural sounds in the auditory brain. As neurons' adaptation to stimulus statistics gradually grows stronger along the auditory pathway, populations of these neurons progressively shift from encoding low-level physical attributes of incoming sounds towards more mean-, contrast-, and noise-independent information about stimulus identity. The result is a major computational step towards the context-invariant, categorical sound representations that are seen in higher areas of AC.

## Materials and Methods

### Animals and Physiology

All animal procedures were approved by the local ethical review committee and performed under license from the UK Home Office.

Extra-cellular recordings were performed in medetomidine/ketamine-anesthetized ferrets. Previous work has shown that this does not affect the contrast adaptation properties of cortical neurons [10]. Full surgical procedures for cortical recordings (primary auditory cortex and anterior auditory field), spike-sorting routines, unit selection criteria, and sound presentation methods (diotic, earphones, 48828 kHz sample rate) are provided in ref. [12]. Surgery for IC recordings were performed as in ref. [55]. Recordings were made bilaterally in both locations.

The AN was simulated using the complete model of Zilany et al. [27]. We generated spiking responses from 100 fibers at a 100 kHz sample rate, with the same distribution of center frequencies (CFs) and spontaneous rates (SRs) as in that paper (see section “AN Model” below); *n* = 85 fibers were used based on reliably evoked responses to the natural stimuli [10],[12].

### Stimuli

Four natural sound segments were presented (forest sounds, rain, female speech, male speech sped up by 50%), with a combined duration of 16 s, to 5 animals (IC, 2 animals, *n* = 80 units; AC, 3 animals, *n* = 124 units). For each sound, noise tokens were synthesized with the same power spectrum and duration, and mixed with the original source. The amplitudes of the source and noise were scaled so that the SNR was 20 dB for the clean condition, and 10/0/−10 dB for the noisy conditions, with a fixed root-mean-square (RMS) level of 80 dB SPL. The “clean” condition was therefore high-SNR, but not entirely noise-free; this was necessary to keep its (log)-spectrogram bounded from below at reasonable values. Fifty unique noise tokens were generated for each sound and each SNR. All sounds included 5 ms cosine ramps at onset and offset. The set of stimuli were presented in random order, interleaved with ∼7 min of DRC stimulation. DRCs were constructed from tones spaced at 1/6-octave intervals from 500 Hz to 22.6 kHz; these changed in level synchronously every 25 ms. Tone levels were drawn from uniform distributions with a mean 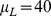 dB SPL, and halfwidths of 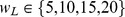 dB. Responses to these DRCs informed the analysis in Figure 8B.

The analysis in Figure 5A–F was from DRCs presented to a further 6 animals (IC, 3 animals, *n* = 136 units; AC, 3 animals, *n* = 76 units); these procedures were as described in ref. [12]. Here, tones were 1/4-octave spaced, and tone-level distributions had 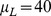 dB SPL and 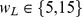 dB. Approximately 30–60 min of DRCs were presented during each penetration. Stimuli in Figures S7 and S8 were presented to 2 animals (IC) and 4 animals (AC).

### AN Model

We simulated the AN using the phenomenological model of Zilany et al. [27]. We chose the Zilany model because it captures many physiological features of the AN responses to simple and complex sounds, including middle-ear filtering, cochlear compression, and two-tone suppression. It does not explicitly model the action of the olivocochlear bundle, such as the medial olivocochlear reflex, which modulates cochlear gain during periods of high-amplitude stimulation [56] and may therefore improve the audibility of transient sounds, such as tones or vowels, in noise [57],[58]. However, it does capture the adaptation of AN responses to the mean level of a sound as experimentally measured in the cat AN [36],[59].

We used the full AN model as provided in the authors' code, including the exact (rather than approximate) implementation of power law adaptation. We simulated 100 AN fibers, using the same distribution of CFs and SRs that the authors used in that paper, based on previous physiological data [60]. Of the 100 fibers, 16 were low SR, 23 were medium SR, and 61 were high SR. For each SR, fibers had log-spaced CFs between 250 Hz and 20 kHz.

We ran three controls on this model. First, we tested whether there was a difference in the results from low, medium, or high SR fibers, and found little to no difference between the metrics presented in the main text. Second, Zilany et al. present both an exact and an approximate implementation of power law adaptation; we therefore simulated both and found that the two implementations produced very similar results.

Finally, the adaptation built into the model allows past stimulation history to affect current responses. We therefore tested whether the decoder results changed as we increased the length of preceding stimulation. To do this, we simulated the stimulus presentation sequences used during physiological recordings, where natural sounds were played back-to-back (with a 100 ms silence between sounds). The stimuli were presented in pseudorandom order, as in physiology experiments. As the time and memory complexity of the sAN simulation algorithm grows exponentially with stimulus length, the longest sequences we were able to present in reasonable time were four sounds (i.e., 16 s) in duration. Next, we selected the responses to either the first, the second, the third, or the fourth sound in each sequence. The first set of responses were generated with 0 s of preceding stimulation; these were discarded to avoid unstable initial behavior. We considered each of the remaining sets of responses: the second set, with an average of 4 s of preceding stimulation; the third, with an average of 8 s; and the fourth, with an average of 12 s. Using this schema, we simulated three entire sAN populations and calculated the relevant decoder metrics for each. There was very little difference between the values of the metrics in Figure 7D–E when the amount of preceding stimulation was varied between 4 and 12 s. We were therefore confident that the simulated adaptation had reached a steady state. Data in the main text are from the fourth set of responses; these are simulated with an adaptation “memory” of 12 s of natural stimulation.

### KL Divergence Calculation

To measure how the distributions of units' responses changed with the addition of noise (Figure 2B), we performed the following analysis for each unit. We began with the trial-averaged, time-varying firing rates evoked over the stimulus ensemble for each SNR (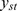, where 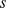 is SNR and 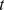 is time), at a 5 ms resolution. We scaled these firing rates relative to the maximum firing rate produced by that unit in the 20 dB SNR condition: 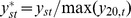. We then approximated the distributions 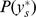 for each SNR 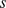, by binning 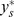 at a resolution (bin size) of 0.01, and using a maximum 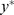 of 2 (enforced for consistency; no 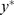 ever exceeded this value). The counts in each bin were augmented by a value of 0.5 (generally about 2%–10% of the observed count; equivalent to using a weak Dirichlet prior with a uniform base measure 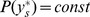); this ensured that the results remained finite. We then normalized the counts to have unitary sum. Finally, we computed the Kullback–Leibler divergence between 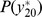 and 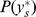, with values shown in Figure 2B.

### Noise Independence Calculation

To assess how the stimulus-conditioned responses depended on the level of background noise, we calculated a mutual information (MI)-based measure for each unit (Figure 2C). For each background-noise condition (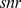), we labeled the stimulus in each time bin with an index, 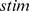, using the same 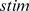 indices across SNRs. We then calculated the (bias-corrected) MI between the unit's evoked response distributions, 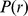, and the 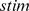 index, 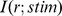, and the MI between 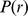 and both the 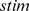 index and the 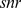, 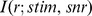. Bias-corrections were performed by shuffling labels [61]. The ratio between these respective quantities measures the proportion of the response entropy that can be reduced by knowing the 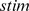 index, as compared with knowing both the 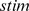 index and the 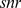. If the responses are statistically independent of the noise, then 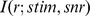 should equal 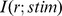, as knowing the 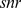 adds no further information. Consequently, a value of 1 means that the response distribution contains information about the underlying sound stimulus but not the level of background noise; lower values mean that the information about the underlying sound stimulus is more SNR-dependent.

### Estimating Contrast-Dependent Gain Changes

To measure how the slope of units' nonlinearities changed as the contrast of the DRC stimuli changed (Figures 5D and 8B), we used the following process. As described in the section “Stimuli” above, units in Figure 5D were stimulated with DRCs used in a previous study [12]. We considered only data from the two uniform contrast conditions in that study—that is, DRC segments where all tone levels were drawn from a distribution with 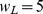 dB (i.e., 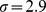 dB), or where all tone levels were drawn from a distribution with 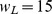 dB (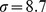 dB). We fitted the following nonlinearity to this dataset:
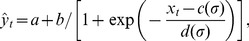
(1)

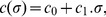
(2)

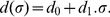
(3)The reported values of 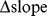 are given as percentages; this is the ratio:
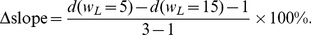
(4)


Thus 0% indicates no slope changes, and 100% indicates perfect compensation for stimulus contrast. It is also possible under this metric that 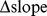 can exceed 100%: this indicates that the unit's gain change was even stronger than was necessary to compensate for the changes in contrast.

The units in Figure 8B were stimulated with a different set of DRCs. These had tone-level distributions with half-widths drawn from 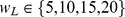 dB (and 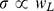 as above). We fitted the same contrast-dependent nonlinearity as above (Equations 1–3). Here, since a broader range of contrasts was used, the reported values of 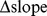 are given as:
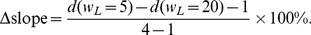
(5)


There were no significant differences between the measures in Equations 4 and 5.

### Estimating Contrast-Dependent Changes in Coding (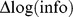)

As the contrast of DRC stimuli changed, units' output nonlinearities predominantly changed their gain (as in Figure 5B). Some units' output nonlinearities also showed other adaptive shifts (examples in Figure S2). To quantify the overall effect of contrast-dependent changes to output nonlinearities, we constructed a measure of how these adaptive shifts change the amount of information a unit's firing rate carries about the ongoing stimulus (Figure 5F).

As above (see “Estimating Contrast-Dependent Gain Changes”; Figures 5D and 8B), we limited our analysis for each unit to data from the two uniform contrast conditions. For each unit, we fitted individual output nonlinearities for the two conditions (these are the blue and red curves shown in Figure 5B and Figure S2A); we denote these two curves as 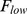 and 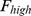, respectively:
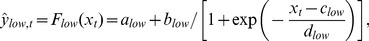
(6)

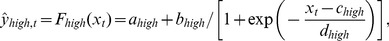
(7)where 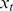 is the STRF-filtered DRC for that unit. Unlike in the previous section, these two nonlinearities were not constrained to have the same values of 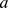 and 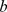.

For sigmoidal 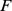, and Poisson spiking, the Fisher information conveyed by the unit about 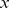 is:
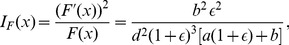
(8)


Where 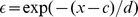.

Using these equations, we estimated the expected 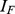 over the low contrast distribution of stimuli for both 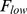 and 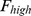. We generated 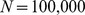 samples of 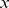 values from the low contrast distribution (by filtering a long, low contrast DRC through the STRF) and calculated the expectations 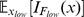 and 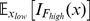 over these samples. Finally, we defined:
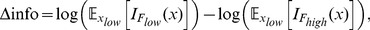
(9)where the logarithm removes the dependency on the maximum firing rate. Thus, this measure estimates how much more Fisher information a unit carries about low contrast stimuli when it is adapted to low contrast stimulation, compared with when it is adapted to high contrast stimulation.

### Population Decoding

Log-amplitude spectrograms of natural sounds were computed with 256 frequency bins (0.1–24 kHz) and downsampled to 5 ms time resolution. Neuronal responses were binned at 5 ms resolution to match the resolution of the spectrograms. Responses to 40 randomly selected repeats of the clean sound were set aside as a training set for the decoder.

We decoded the stimulus spectrogram from population responses using a dictionary approach. We made the following assumptions: (1) the responses of pairs of units, or of a given unit at two different times, were conditionally independent given the stimulus; (2) the expected firing rate of unit 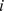 in time bin 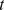 was a function of the recent history of stimulation—that is, of the spectrogram segment 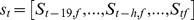 (where 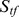 is the full sound spectrogram, 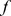 is frequency, and 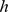 is a history index, covering 20 bins—i.e., 100 ms); and (3) the observed firing rate of unit 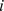 at time 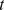, 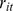, was the result of an inhomogeneous Poisson process, with 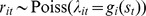 for some function 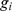. Rather than attempting to parameterize 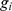, we obtained maximum a posteriori estimates of 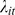 from the 40 repeats of the training data, using a conjugate prior 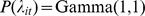. This prior ensured that 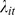 was always greater than 0.024; if 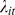 were allowed to drop to 0, the decoder results would be skewed by units with very low average firing rates.

Inference consisted of calculating, for each time bin 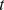, the posterior distribution over spectrogram segments 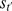, which could have produced the responses in that bin. Because only 16 s of unique training stimuli were presented (i.e., only approximately 3,200 spectrogram segments), the log posterior over this reduced set of elements, 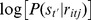, could be fully computed from the responses of each unit 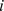, time bin 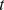, and repeat 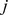 (via a uniform prior over the presented 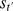, assumption (3), and Bayes' rule), and then summed across units and repeats by assumption (1). A single estimate of 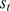 was then produced from the posterior mean, 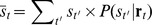.

Finally, it was necessary to integrate the successive binwise estimates of recent spectrogram history, 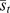, into a single decoded spectrogram, 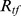. This we achieved by convolution with a kernel: 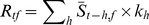. Given typical neural integration dynamics, we used exponential kernels, 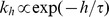. Optimal 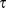 values were found at 25/35/100 ms for sAN/IC/AC, by maximizing 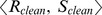 as a function of 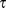 over a validation data set. The choice of 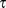 nevertheless had very little impact on decoder metrics (Figure S9).

Spectrograms were decoded from responses to the remaining 10 repeats of the clean sounds, as well as from responses to 10 repeats from each of the noisy sound presentations.

To compare spectrograms 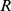 and 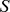, we calculated the mean square error (MSE) between the two, as 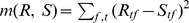. We scaled these values relative to a “prior MSE,” 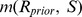, where 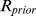 is the spectrogram decoded from the prior distribution over 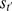, such that 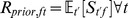. The prior MSE gives the error when a decoder has no neural responses to decode, so all stimuli in the dictionary are equally likely. We defined the decoded spectrogram similarity metric as 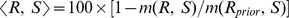.

As described in the main text, and in Figure 7A, the absolute fidelity of these reconstructions, 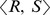, differed between sAN, IC, and AC. Our interest was not, however, in these absolute quantities, but rather in how the reconstruction fidelity changed within a location when noise was added. We therefore calculated, for each location, the degradation of reconstruction fidelity relative to the low noise condition, via the normalized metrics, 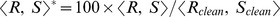. This uses each low noise condition as an internal control for each location. These metrics were stable with population size (Figure S10).

Metrics could take negative values when reconstructions were very poor; this occurred when MSEs were worse than the prior MSE. For Figure 8B, sAN values of 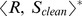 were adjusted for low BI: we removed the discrepancy between inferred and actual spectrogram means via an adjusted MSE, 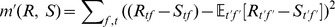. Unadjusted data are shown in Figure S6.

Error bounds on similarity metrics were obtained by bootstrapping. We subsampled units from the respective populations 50 times over and parameterized the bootstrapped statistics with Gaussians.

Several features of this decoder are worth particular mention.

We assumed that neural responses were conditionally independent given the stimulus. Note that this is not an assumption that neurons are wholly independent of one another (e.g., that STRFs did not overlap, or that signal correlations were 0), but rather that trial-to-trial correlations were not relevant to stimulus coding (i.e., that noise correlations were 0). Thus, though we simultaneously recorded an average of four neurons at a time per electrode penetration, we grouped all nonsimultaneously recorded data together, and discarded the trial labels. Although noise correlations do exist among auditory neurons [62],[63], to our knowledge, there are few existing studies that successfully take this coordinated variability into account to improve high-dimensional stimulus reconstruction [43]–[45]. Here, we made the assumption of conditional independence for two reasons: (1) since our AN model had no correlated noise source, we wished to put the decoders from the three locations on an equal footing; (2) more importantly, ignoring noise correlations rendered inference far more tractable. It is nevertheless likely that, using more sophisticated decoders, absolute reconstruction fidelity would improve with noise correlations taken into account [64]; this has been found to be the case in recent decoding studies attempting stimulus categorization [65],[66]. In building such models for reconstruction, it would also be important to address the empirical question as to how correlations between auditory neurons change as background noise is introduced into a sound [65],[67].

Our decoder was trained on a limited set of signals, namely 40 repeats of 16 s of “clean” (20 dB SNR) sound stimulation. As a result, the output of the decoder was restricted to convex combinations of spectrogram segments from the training signals (i.e., a dictionary). The decoder was therefore not a general-purpose algorithm. Nevertheless, by design, the noisy spectrograms lay within the reconstruction space. In particular, decoding with no information (or when the decoder rates each stimulus segment as equally likely) produces the spectrogram of the added noise.

It is worth emphasizing that the decoder therefore had implicit knowledge of the clean signals' inherent structure, via the dictionary of spectrogram segments. In particular, this amounts to a prior on the spectrogram correlations over a 100 ms history. In general, incorporating such prior knowledge has been demonstrated to improve the performance of spectrogram reconstruction algorithms [43],[44]; conversely, such a strong prior as a dictionary reduces the ability to extrapolate to signals that lack this structure. Our emphasis in this work, therefore, is not on the gross performance of the decoder, but on how well its assumptions about the respective populations' encoding schemes remain robust across noise conditions. In this respect, a high similarity between 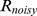 and 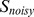 indicates that a population encodes the noise in a noisy stimulus much like the signal in the clean stimulus (which the decoder is trained to decode). On the other hand, a high similarity between 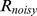 and 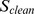 indicates that a population tends to encode the sound features that are common between the clean and noisy sounds.

Finally, it is an empirical question beyond the scope of this article as to whether the decoded responses would maintain these properties with more structured sources of background noise, or those that lay outside the training set of the decoder.

## Supporting Information

Figure S1
**Increasing independence of response distributions to background noise level cannot be explained by increased modulation filtering.** This figure shows a simulated experiment designed to test whether the results in Figure 2 could be explained by changes in the temporal integration properties of neurons in the auditory pathway. We constructed populations of model auditory neurons, simulated their responses to the natural sounds presented in the main text, and performed the same analysis as in Figure 2. The populations were identical except for the parameter 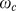, defining the temporal integration properties of the model neurons. Further details follow, but in brief, (A) shows a general schematic for how the model neurons process sound stimuli, (B) illustrates how 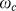 affects input signals in the model, and (C) is a direct analogue of Figure 2C, using the model neurons. (A) Model of auditory neurons used in the simulation. This comprises two stages. The first stage is a simple model of cochlear filtering. We began with the pressure waveforms of the natural sounds used in the main text. We simulated frequency-selective cochlear channels by filtering the sound waveforms through a gammatone filterbank. This was implemented as a set of 50 IIR gammatone filters [68], using the Brian simulator [69] in Python. Filter CFs were ERB-spaced between 250 Hz and 20 kHz, as in ref. [70]. We next extracted the amplitude envelope of each filter output, via the magnitude of the Hilbert transform. We then applied a 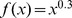 compressive nonlinearity to envelopes to approximate the amplitude compression that occurs at the cochlea [71]. In the second stage, we constructed populations of model auditory neurons, based on the output of the 50 cochlear channels. Populations were defined by the choice of a single parameter, 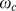, which characterizes the temporal integration properties of the model neurons in each population. We assumed that each auditory neuron within a model population received input from only one peripheral channel. As a simple approximation of how the modulation-following characteristics of neurons change as one ascends the auditory pathway [1], we low-pass filtered the inputs to these model neurons, using an 8th-order Chebyshev Type I low-pass filter, with a cutoff frequency chosen from either 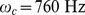 (to model AN neurons, denoted here as mAN), 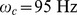 (to model IC neurons, denoted here as mIC), or 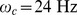 (to model cortical neurons, denoted here as mAC). Next, we passed the modulation-filtered input signal for each neuron, 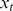, through a sigmoidal output nonlinearity. The output of this stage was a time-varying firing rate, 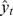, from which we generated spike trains via an inhomogeneous Poisson process. Thus, for each model location (defined by 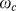), we generated a set of spike data of the same form as that used in the main text. The model used here is equivalent to a linear-nonlinear-linear-nonlinear-Poisson (LNLNP) forward model. The gammatone filters, Hilbert envelope, and compressive nonlinearity cast the time-varying pressure signal into a 50-dimensional time series via a LN process (the first LN of the LNLN model). The second linear (L) stage was similar to that used in a STRF model: each model neuron collapsed this high-dimensional signal down to a one-dimensional time-series via a convolution with a spectro-temporal kernel. We used simple kernels: these were separable in frequency and time, sparse in the frequency domain (the weight was nonzero for only one frequency channel), and modulation low-pass in the time domain. The final nonlinear (N) stage was provided by a point nonlinearity. (B) A 1.5 s segment of 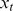, the “within-channel intensity” (i.e., STRF-filtered input signal) of a model auditory neuron as described in (A). These were produced from a cochlear filter with a CF of 1.3 kHz, together with AN-, IC-, and AC-like modulation filtering as simulated from the model in (A). These panels parallel Figure 3A, showing the within-channel intensity from a clean (20 dB SNR) sound (lower lines in the left panels), and that from a noisy (0 dB SNR) version of the same sound (upper lines). The mAC neuron is more modulation low-pass; fluctuations in sound intensity introduced by the noise have less energy for the mAC neuron than for the mAN fiber. (C) Statistical independence of stimulus-conditioned response distributions 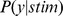 to the background noise level, measured from the populations of model neurons. This panel is a direct analogue of Figure 2C. Median values of noise independence for mAN/mIC/mAN were 0.80/0.80/0.83. Since the only factor that differentiates the mAN, mIC, and mAC populations from each other is the modulation cutoff frequency, 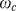, this estimates that increased modulation filtering along the auditory pathway is responsible for about a third of the effect observed in the measured data in Figure 2C. The larger differences between auditory centers observed in the main text could be obtained by simulating increased 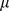
- and 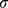-adaptation along the auditory pathway, as in Figure S5 (unpublished data).(TIFF)

Figure S2 **Further examples of adaptation to contrast, as shown in** **Figure 5B–C** **.** In addition to the general trend of an increasing slope of the nonlinearity with contrast, some sAN fibers (Examples 1 and 2) underwent small shifts in mean level at lower contrast; greater effects were seen in some IC units (Examples 4 and 5). Some IC units showed other contrast-dependent changes to nonlinearities, including horizontal shifts (Example 1) and changes in saturation points (Example 3). While more complex models of contrast-dependent changes to nonlinearities were sometimes needed to characterize the behavior of IC neurons (such as the more general classes of contrast kernel models described in ref. [12]),changes in slope for IC units were, overall, smaller than in cortex, but larger than in the sAN.
(TIFF)

Figure S3
**Fitted time constants for gain control at different levels of the auditory pathway.** These time constants were obtained using the same stimuli and procedure as previously documented [12]. After a change in the spectral pattern of contrast of a DRC, the gain of IC and cortical units' nonlinearities changed with an approximately exponential time course, with median time constants of 35 ms in IC and 117 ms in AC. Contrast-dependent gain changes were generally weak or nonexistent in the sAN, with estimated time constants being below 25 ms (and hence not detectable with this method). Pairwise differences significant at 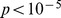 (rank-sum tests).(TIFF)

Figure S4
**The more uniform coverage of frequency space by the simulated AN population does not explain the decoding results in the main text.** (A) Histogram of best frequencies of units in each location. (B, C) The more uniform frequency coverage by the population of sAN fibers, compared with that of the measured IC and cortical populations, could not explain the differences in normalized decoder performance shown in Figure 7D–E. Here, we halved the sAN population in size, keeping only the simulated fibers with higher CFs (>2 kHz). This produced near identical values of 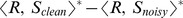 (B) and 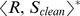 (C) to the full sAN population. While these relative metrics remained unaffected, the absolute performance of the decoder for the clean sound (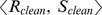) was lower for the high-CF subpopulation than the full sAN population (not shown). This is consistent with the trends shown in Figure 7A: since the high-CF subpopulation contained only 42 simulated fibers (rather than the full 85), there was less information available for inference. However, 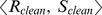 for the high-CF subpopulation was lower than that predicted by Figure 7A: subpopulations of 42 randomly selected fibers (i.e., with more uniform coverage of the spectrum) yielded values of 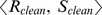 that were on average 10 percentage points higher than the high-CF subpopulation. Thus we can conclude that the greater coverage of the frequency spectrogram by the population of simulated AN fibers, compared with that of the measured IC and cortical populations, contributes to the better absolute decoder performance for the clean sound (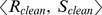) in the sAN.(TIFF)

Figure S5
**Simulation of how both temporal integration and adaptation affect the population encoding of complex sounds, with and without background noise.** This figure shows simulated experiments designed to test whether the results in Figure 7D and 7E could be explained by changes in the temporal integration and/or adaptation properties of neurons in the auditory pathway. As in Figure S1, we constructed populations of model auditory neurons, simulated their responses to the natural sounds presented in the main text, and performed the same decoding analyses as in the main text. The simulation was similar to that performed in Figure S1, and thus followed the same schema as in Figure S1A. However, Figure S1 only considered populations of neurons that differed in their temporal integration properties. Here, we simulated populations that also differed in the strength of their adaptation to stimulus statistics. We constructed populations of model neurons that were identical to each other, except for the value of three parameters: 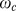, defining the temporal integration properties of the model neurons (as in the simulations in Figure S1); 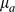, defining the strength of the model neurons' adaptation to the mean intensity; and 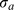, defining the strength of the model neurons' adaptation to the stimulus contrast. Varying these parameters allowed us to test hypotheses about the factors underlying the results in Figure 7D–E. For each population, the values of 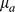 and 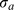 affected the operation of each neuron's sigmoidal output nonlinearity. The shapes of these output nonlinearities were allowed to vary as a function of stimulus statistics, in order to impart adaptation to the neuron. Thus, for each model location (defined by 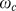), and each set of adaptation parameters (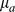 and 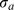), we generated a set of spike data of the same form as that used in the main text. Further details follow, but in brief: (A) illustrates how 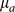 and 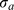 affect the output nonlinearities of neurons in the model; (B) shows the results of fitting these parameters to model populations under different constraints, and compares the performance of the models (symbols) directly with the observed data described in the main text (histogram bars; cf., Figure 7D–E). (A) Adaptive output nonlinearities used in the model. Neural responses were simulated as in Figure S1A, except that each neuron's output nonlinearities was changed for each of the 16 presented stimuli (4 unique sounds ×4 SNRs). The 3×3 grid of panels shows how different values of the parameters 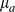 and 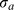 change the way a model neuron's output nonlinearities depend on stimulus statistics. The two lower panels show stimulus statistics (as in Figure S1B) for two example sounds (red and blue), and for the ensemble of all sounds presented. The parameters 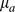 and 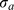 quantify the degree to which output nonlinearities changed as a function of sound statistics (respectively, as a function of the mean of the distribution of within-channel intensities and of the standard deviation). We modeled changes in the neurons' stimulus–response relationships based on observations from experiments using synthetic stimuli (Figures 5, S5, S6, and S10; see also previous work in refs. [9],[10],[12],[20],[36],[72]). These data suggest that when the stimulus 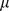 and 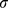 change, auditory neurons' output nonlinearities undergo compensatory shifts. This includes horizontal shifts due to changes in mean level (Figures S7 and S8) and slope changes due to changes in stimulus variance or contrast (Figures 5 and S2). While other changes to neurons' nonlinearities and/or spectral and temporal integration properties may also change with stimulus statistics (e.g., refs. [9],[16],[20],[36]), we focused here on these two major effects. We used sigmoidal output nonlinearities for all model neurons, of the form 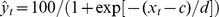, with a maximum firing rate of 100 spikes/s, a minimum of 0, an inflection point at 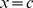, and a natural scale (i.e., inverse gain) of 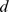. The values of 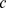 and 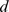 depended on 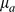 and 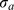, respectively. The extent of 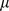-adaptation (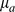) was used to determine whether the parameter c was the same for all 16 sounds (4 sound identities × 4 SNRs; 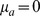), or whether it differed across sounds (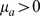). Likewise, the extent of *σ*-adaptation (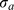) was used to determine the extent to which 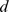 differed across sounds. Thus 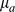 and 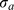 determined how the output nonlinearity changed from sound to sound. Adaptive output nonlinearities for a given model neuron were calculated as follows. We began by calculating the within-channel intensities, 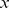 (as illustrated in Figure S1B), for each of the 16 sounds. We denote the distributions of within-channel intensities for these 16 sounds as 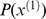; …; 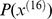, and the distribution of within-channel intensities over the ensemble of all the sounds as 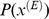. We denote the mean and standard deviation of these distributions as 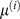 and 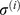, respectively (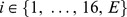). Three of these distributions for the mAC neuron in (B) are illustrated in the bottom two panels of (C). In the bottom-most panel, the gray area shows 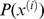, the black dashed vertical line shows 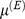, and the thick black horizontal line shows 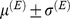. In the second bottom panel, red and blue areas (and lines) show the respective distributions from two individual sounds within the ensemble. For brevity, we refer to these two examples here as the red and the blue sound. Next, the nonlinearity parameters 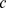 and 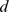 were calculated for sound 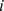 as:
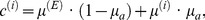
(10)

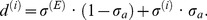
(11)The top left grid panel in (A) shows the output nonlinearity for a model neuron with no 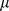
- or 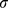
- adaptation—that is, 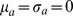. Here, 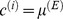 and 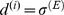, which are both independent of 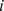. This model neuron thus has a fixed output nonlinearity (black line) that is independent of stimulus statistics. Vertical dashed lines show the means of the distributions 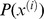 for the ensemble, red, and blue sounds. This fixed output nonlinearity is shadowed in gray for reference in the remaining eight panels in the grid. The bottom left grid panel shows the output nonlinearities for the red and blue sounds for a model neuron with 100% 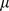-adaptation and 0% 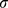-adaptation (i.e., 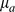 = 1, 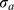 = 0). This neuron has 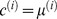, so it adapts its coding for sound 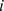 so that the inflection point of its nonlinearity is centered around 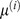. The top right grid panel shows the output nonlinearities for the red and blue sounds for a model neuron with 0% 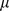-adaptation and 100% *σ*-adaptation (i.e., 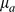 = 0, 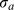 = 1). This model neuron has 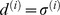, so it adapts its coding for sound 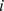 by changing its slope to match the width of 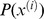. The remaining grid panels show how other example values of 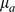 and 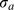 affect output nonlinearities when coding the red and blue sounds. Intermediate values of 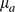 and 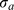 yield only partial adaptations of 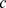 and 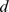 to 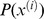. In total, we simulated model neurons with values of 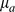 ranging from 0% to 100% in 5% increments, and the same for 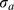; thus, this grid exemplifies only 9 of the 441 pairs of 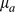 and 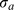 values. (B) Our goal was to determine the extent to which the three factors—differences in modulation filtering (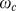), adaptation to the stimulus mean level (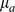), and adaptation to the stimulus contrast (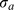)—could account for the observations presented in Figure 7D (the apparent shift from representing 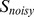 towards representing 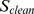) and Figure 7E (the increased noise-tolerance in decoding 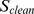). To do so, we determined the values of 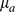 and 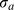 for a model AN population (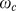 = 750 Hz), a model IC population (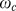 = 95 Hz), and a model AC population (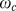 = 24 Hz), which produced representations of natural sounds best matched to the observations in Figure 7D–E. We fitted 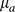 and 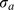 under five different sets of constraints (shown here as separate rows), to test whether and how each of the three parameters (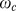, 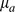, and 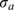) contributed to these results. For each experiment, the observed data from Figure 7D are shown as the histogram bars in the middle column, and the observed data from Figure 7E are shown as the histogram bars in the right column. The symbols in these two columns show the values of these metrics obtained from modeling. The left columns show fitted values of 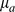 and 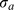, as explained below. As these experiments required extensive simulation, 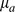 and 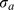 were calculated to 5% precision. We present five experiments here as separate rows. In the first experiment, nonlinearities were fixed (i.e., there was no adaptation; 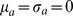). Here, mAN/mIC/mAC populations differed only by their values of 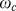. In the second experiment, 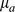 and 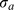 were free to vary, but were each constrained to be identical across the mAN, mIC, and mAC populations (giving a model with two free parameters). As in the first experiment, the three populations differed only in 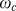. We allowed 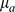 to vary between the three populations in the third experiment (4 free parameters), 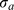 to vary between the three populations in the fourth experiment (4 free parameters), and both to vary across location in the fifth experiment (6 free parameters). In each case, we fitted the free parameters to minimize the total squared error between the 18 data points in Figure 7D and 7E (as obtained from IC and cortical recordings, and from the full AN simulation), and the model populations' values of these metrics. These are shown in middle and right columns of each row (histogram bars show observed values; symbols show model values). The best fit values of 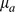 and 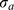 are shown in the left column. First row, in the absence of adaptive coding, differences in modulation tuning could not account for the 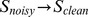 shift, nor the increased noise-tolerance of 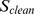 coding. These data do show an important reference: in the absence of adaptation, populations of auditory neurons would encode 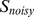 rather than 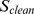 (middle panel). Second row, in the presence of adaptive coding, differences in modulation tuning partially contribute towards increased noise-tolerance of 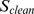 encoding from periphery to cortex, but are not sufficient to explain the 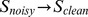 shift. Third row, allowing the strength of adaptation to stimulus mean (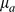) to take different values for the model AN, IC, and AC populations was sufficient to explain the 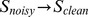 shift, but not the increased noise-tolerance of 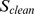 encoding. Fourth row, allowing the strength of adaptation to stimulus contrast (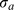) to take different values for the mAN, mIC, and mAC populations was sufficient to explain the increased noise-tolerance of 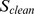 encoding, but not the 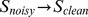 shift. Bottom row, allowing both the strength of adaptation to stimulus mean and stimulus contrast to change for each model population can explain the results observed in Figure 7D and 7E. This analysis predicts that both the strength of adaptation to the stimulus mean (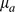) and the strength of adaptation to its contrast (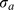) should increase from the AN to the IC to the cortex.(TIFF)

Figure S6
**Adjusted** 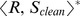 **for sAN units in** **Figure 8B** **.** The results of Figure 8B show the relationship between the strength of *σ*-adaptation and the noise-tolerance of 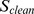 encoding. However, 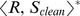 is also affected by BI (Figure 8A). Because the sAN units had low BI (Figure 4B), decoding the responses of the sAN population to noisy sounds produced spectrograms that included the noise present in 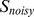 but not 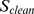 (Figure 6); as a result, 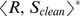 was even lower for the sAN. Therefore, to elucidate the relationship between 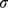-adaptation and the noise-tolerance of 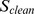 encoding, we compensated for the low BI of sAN units in that figure. As described in Materials and Methods, this involved using a baseline-corrected similarity metric, which ignored the difference in mean between the decoded and clean spectrograms. Here, we show the effect of that compensation on 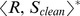. Pluses show the uncorrected metric for the sAN; stars show the corrected metrics as in Figure 8B. The correction had little to no impact on 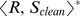 for IC and cortical subpopulations; for the IC and AC data points on this plot, the difference between corrected and uncorrected metrics differed by an average of 0.5% (and hence are not depicted).(TIFF)

Figure S7
**A separate set of experiments characterizing adaptation to the mean stimulus intensity in sAN, IC, and AC neurons.** (A) Schematic of a LN model. In this experiment, we probed auditory neurons using DRC stimuli. As in the experiment presented in Figure 5, these were constructed as superpositions of tones, whose time-varying levels, 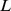, were drawn from particular distributions (shown in B). The transformation of the sound into a time-varying spike rate (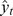) is modeled as a two-stage procedure: first, the sound spectrogram (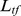; top and bottom; colors denote tone level) is filtered through a linear STRF. This reduces the large dimensionality of the input space to a 1D time-varying signal, 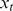. Second, this signal is passed through a sigmoidal output nonlinearity, yielding the firing rate (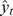). (B) Statistics of the DRCs were controlled by varying the distribution of tone levels, 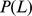. In this set of experiments, the mean (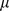) of 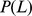 was varied (cf., the experiment shown in Figure 5, where the width of 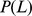 was varied). (C) For each unit, the distribution of STRF-filtered DRCs, 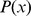, depends on the distributions 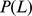 shown in (B). (D) Illustration of a fixed output nonlinearity for an idealized neuron with no adaptation to the mean. The two colors show the portion of the nonlinearity that would be explored by the stimulus distributions shown in (B) and (C). (E) Illustration of two output nonlinearities for an idealized neuron with complete (dynamic-range) adaptation to the mean. This neuron no longer has a single fixed output nonlinearity; rather, the nonlinearity is horizontally shifted to cover the presented range of 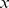 values. (F) Data from example units in each location. These show how output nonlinearities change as the mean tone level (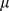) changed. STRFs (insets) range from 0.5 kHz to 22.6 kHz on the frequency (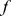) axis, and are shown over only 100 ms of the 200 ms history (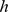) at 25 ms resolution. Colors denote nonlinearities in different mean-level conditions; corresponding distributions 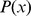 shown below. For the example AN fiber, there is (approximately) a single output nonlinearity that remains relatively unchanged as a function of 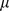; in the example IC and cortical units, output nonlinearities undergo considerable horizontal shifts as a function of 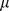. Further examples shown in Figure S8. (G) Nonlinearities in (F), replotted as a function of normalized 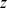 coordinates. 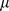-adaptation induces a shift away from the encoding of the unnormalized signal, 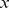, in the periphery, towards the encoding of the normalized signal, 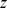, in IC and cortex. (H) Histogram of the degree of 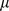-adaptation in each location. This was measured by fitting a single sigmoid for all the output nonlinearities, with a 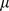-dependent inflection point:
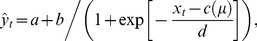
(12)

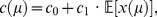
(13)where 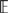 is expectation over the distribution of STRF-filtered signals. Here, 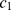 measures the horizontal displacement of the curve. A value of 0% (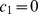) indicates an independent encoding of the unnormalized variable, 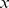. A value of 100% (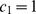) indicates complete compensation for mean level. The median shift was 7% for the simulated AN units (*n* = 85), 101% for the recorded IC units (*n* = 32), and 100% for the cortical data (*n* = 287). The difference between IC and AC was not significant (rank-sum test; *p*>0.5), but the differences between AN and IC/AC were (*p*<10^−6^). As these data were collected from different units from the natural sound study described in the main text, we could not compare the magnitude of the 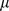-dependent shift in output nonlinearities with the decoder metrics.(TIFF)

Figure S8
**Further examples of adaptation to mean tone level, as shown in Figure S7F–G.** (A) Output nonlinearities for five example sAN fibers (left), five IC units (middle), and five cortical units (right). Insets show units' STRFs, as in Figure 5B. For each example, top panel shows the fitted output nonlinearities for DRCs presented at different mean levels. All DRCs were constructed of pure tones; tones had levels drawn from a uniform distribution with halfwidth 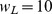 dB, and means of 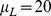 dB SPL (orange), 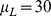 dB SPL (green), 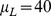 dB SPL (blue), or 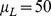 dB SPL (purple). Three to four of these conditions were usually presented for each unit; some IC units were only tested with two 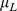 conditions. Using the LN model shown in Figure S7A, the DRC stimuli produced from each of these tone-level distributions are filtered through units' STRFs to produce time-varying signals, 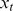. The statistics of 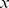 for each condition are a function of the coefficients in the STRF. Thus, the distributions 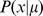 vary from unit to unit in a number of ways. For example, STRFs dominated by a single coefficient (e.g., sAN Example 4, IC Example 1) have more uniform-like 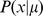, while STRFs with a large number of nonzero coefficients are more Gaussian-like (e.g., most cortical units). Also, the net balance between excitatory (red) and inhibitory (blue) coefficients of the STRF determine how increasing *μ* changes the mean of the distribution 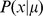. With more excitation in the STRF (most examples), 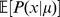 increased for larger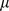; with more inhibition, 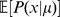 decreased for larger 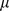 (AC Examples 1, 4, and 5). In a small number of cases, excitation and inhibition were approximately equal (AC Example 2), such that 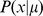 did not change considerably with 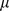. (B) Output nonlinearities for the units in (A), replotted as a function of normalized coefficients, 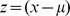, as in Figure S7G. As in Figure 5B–C, output nonlinearities were generally independent of 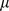 in the sAN, but changed considerably with mean level in the IC and cortex. The trend was such that in these higher stages of the pathway, responses were better described as a function of normalized coefficients. While differences in the shape of nonlinearities often arose in IC and cortex from changing 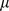 (e.g., IC Example 5, AC Example 3), a simple horizontal shift in nonlinearities usually described a major component of the 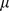-dependent changes.(TIFF)

Figure S9
**Differences in decoder performance were not the result of the time constants used to reconstruct spectrograms.** As described in Materials and Methods, the decoder constructs an estimate of the recent spectrogram history for each 5 ms bin. In order to integrate these successive estimates into a single decoded spectrogram, we convolved the set of estimates with exponential kernels, 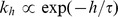, where 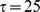 ms for sAN, 35 ms for IC, and 100 ms for AC. Here, similarity metrics as used in the main text are shown for values of *τ* ranging from 5 ms to 100 ms. As in Figure 7, shaded regions show 95% confidence intervals. Filled circles show the *τ* values used in the main text; these were chosen to maximize 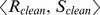 for each location. However, values of 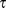 between 25 ms and 100 ms produced very similar results for all locations.(TIFF)

Figure S10
**Stability of metrics with increasing population size.** In Figure 7A, we show that the values of the decoder metric 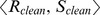 generally increased as more units were included in the analysis. Here, we show how the normalized metrics (A) 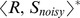, (B) 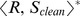, and (C) 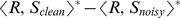 converged to stable values as the number of units included in the analysis was increased. Thus, the differences across location in the normalized decoder metrics shown in Figure 7D–E are not the result of differences in the absolute fidelity of the decoding.(TIFF)

Table S1
**Contributions of increasing BI and CI along the auditory pathway to the results in** **Figure 8** **.** In Figure 8A, we demonstrate that the shift from 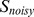-representations in the sAN population to 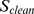-representations in the AC population can largely be explained by an increase in neurons' BI along the auditory pathway. In Figure 8B, we demonstrate that the increasing robustness of 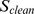 encoding can largely be explained by an increase in neurons' contrast invariance along the auditory pathway. This table documents the statistics for these two figures (A for Figure 8A; B for Figure 8B). The percentages shown quantify the contributions of BI and CI toward explaining the differences between the decoder metrics across locations. The values are relative effect sizes within a general linear model. They were calculated by fitting a set of multiple linear regression models (ANCOVA) to (A) the data points in Figure 8A (where the decoder metric is 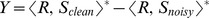) and (B) Figure 8B (where the decoder metric is 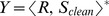). The first row of the table considers only the differences between sAN and IC data (for each of A and B, *n*  =  24 data points  =  3 SNRs × 4 subpopulations × 2 locations); the second row considers only the differences between IC and AC data (24 data points); while the third row considers the differences across all three locations (36 data points). To calculate relative effect sizes for (A), we fitted the following four linear models:
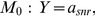
(14)

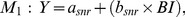
(15)

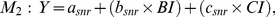
(16)

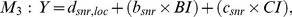
(17)where 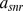, 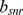, 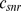, and 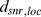 are categorical variables. Model 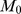 is the reference model; model 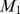 adds BI as an explanatory variable, 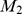 adds CI, and 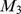 captures across-location differences that remain unexplained by BI and CI. Denoting the residual variance for model 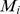 as 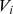, the relative effect size of BI was calculated as 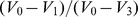. The relative effect size of CI was calculated as 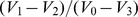. The unexplained portion was calculated as 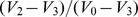. The procedure for calculating relative effect sizes for (B) was identical, except the order of adding BI and CI to the multiple linear regression model was reversed.(PDF)

## Abbreviations

AC: auditory cortex
AN: auditory nerve
BI: baseline invariance
CDF: cumulative distribution function
CF: center frequency
dB: decibels
DRC: dynamic random chord
IC: inferior colliculus
KL: Kullback–Leibler
LN: linear–nonlinear
mAC: model auditory cortex
mAN: model auditory nerve
mIC: model inferior colliculus
MSE: mean square error
MTF: modulation transfer function
PSTH: peri-stimulus time histogram
RMS: root mean square
sAN: simulated auditory nerve
SNR: signal to noise ratio
SPL: sound pressure level
SR: spontaneous rate
STRF: spectro-temporal receptive field

## References

[1] Joris PX, Schreiner CE, Rees A (2004) Neural processing of amplitude-modulated sounds. Physiol Rev 84: 541–577. 15044682 10.1152/physrev.00029.2003

[2] Young ED (2008) Neural representation of spectral and temporal information in speech. Philos Trans R Soc Lond B Biol Sci 363: 923–945. 17827107 10.1098/rstb.2007.2151 PMC2606788

[3] Schreiner CE, Froemke RC, Atencio CA (2011) Spectral processing in auditory cortex. In: Winer JA, Schreiner CE, editors, The auditory cortex, Springer. pp. 275–308.

[4] Formisano E, Martino FD, Bonte M, Goebel R (2008) “Who” is saying “what”? brain-based decoding of human voice and speech. Science 322: 970–973. 18988858 10.1126/science.1164318

[5] Okada K, Rong F, Venezia J, Matchin W, Hsieh IH, et al (2010) Hierarchical organization of human auditory cortex: evidence from acoustic invariance in the response to intelligible speech. Cereb Cortex 20: 2486–2495. 20100898 10.1093/cercor/bhp318 PMC2936804

[6] Chang EF, Rieger JW, Johnson K, Berger MS, Barbaro NM, et al (2010) Categorical speech representation in human superior temporal gyrus. Nat Neurosci 13: 1428–1432. 20890293 10.1038/nn.2641 PMC2967728

[7] Ding N, Simon JZ (2012) Emergence of neural encoding of auditory objects while listening to competing speakers. Proc Natl Acad Sci 109: 11854–11859. 22753470 10.1073/pnas.1205381109 PMC3406818

[8] Ding N, Simon JZ (2013) Adaptive temporal encoding leads to a background-insensitive cortical representation of speech. J Neurosci 33: 5728–5735. 23536086 10.1523/JNEUROSCI.5297-12.2013 PMC3643795

[9] Nagel KI, Doupe AJ (2006) Temporal processing and adaptation in the songbird auditory forebrain. Neuron 51: 845–859. 16982428 10.1016/j.neuron.2006.08.030

[10] Rabinowitz NC, Willmore BD, Schnupp JW, King AJ (2011) Contrast gain control in auditory cortex. Neuron 70: 1178–1191. 21689603 10.1016/j.neuron.2011.04.030 PMC3133688

[11] Sharpee TO, Nagel KI, Doupe AJ (2011) Two-dimensional adaptation in the auditory forebrain. J Neurophysiol 106: 1841–1861. 21753019 10.1152/jn.00905.2010 PMC3296429

[12] Rabinowitz NC, Willmore BDB, Schnupp JWH, King AJ (2012) Spectrotemporal contrast kernels for neurons in primary auditory cortex. J Neurosci 32: 11271–11284. 22895711 10.1523/JNEUROSCI.1715-12.2012 PMC3542625

[13] Rees A, Møller AR (1987) Stimulus properties influencing the responses of inferior colliculus neurons to amplitude-modulated sounds. Hear Res 27: 129–143. 3610842 10.1016/0378-5955(87)90014-1

[14] Rees A, Palmer AR (1989) Neuronal responses to amplitude-modulated and pure-tone stimuli in the guinea pig inferior colliculus, and their modification by broadband noise. J Acoust Soc Am 85: 1978–1994. 2732379 10.1121/1.397851

[15] Krishna BS, Semple MN (2000) Auditory temporal processing: responses to sinusoidally amplitude modulated tones in the inferior colliculus. J Neurophysiol 84: 255–273. 10899201 10.1152/jn.2000.84.1.255

[16] Kvale MN, Schreiner CE (2004) Short-term adaptation of auditory receptive fields to dynamic stimuli. J Neurophysiol 91: 604–612. 14762146 10.1152/jn.00484.2003

[17] Lesica NA, Grothe B (2008) Efficient temporal processing of naturalistic sounds. PLoS ONE 3: e1655 doi:10.1371/journal.pone.0001655 18301738 10.1371/journal.pone.0001655 PMC2249929

[18] Blake DT, Merzenich MM (2002) Changes of AI receptive fields with sound density. J Neurophysiol 88: 3409–3420. 12466457 10.1152/jn.00233.2002

[19] Valentine PA, Eggermont JJ (2004) Stimulus dependence of spectro-temporal receptive fields in cat primary auditory cortex. Hear Res 196: 119–133. 15464309 10.1016/j.heares.2004.05.011

[20] Dean I, Harper NS, McAlpine D (2005) Neural population coding of sound level adapts to stimulus statistics. Nat Neurosci 8: 1684–1689. 16286934 10.1038/nn1541

[21] Watkins PV, Barbour DL (2008) Specialized neuronal adaptation for preserving input sensitivity. Nat Neurosci 11: 1259–1261. 18820690 10.1038/nn.2201

[22] Bar-Yosef O, Rotman Y, Nelken I (2002) Responses of neurons in cat primary auditory cortex to bird chirps: effects of temporal and spectral context. J Neurosci 22: 8619–8632. 12351736 10.1523/JNEUROSCI.22-19-08619.2002 PMC6757805

[23] Bar-Yosef O, Nelken I (2007) The effects of background noise on the neural responses to natural sounds in cat primary auditory cortex. Front Comp Neurosci 1: 3. 10.3389/neuro.10.003.2007 PMC2525935 18946525

[24] Escabí MA, Miller LM, Read HL, Schreiner CE (2003) Naturalistic auditory contrast improves spectrotemporal coding in the cat inferior colliculus. J Neurosci 23: 11489–11504. 14684853 10.1523/JNEUROSCI.23-37-11489.2003 PMC6740949

[25] Shetake JA, Wolf JT, Cheung RJ, Engineer CT, Ram SK, et al (2011) Cortical activity patterns predict robust speech discrimination ability in noise. Eur J Neurosci 34: 1823–1838. 22098331 10.1111/j.1460-9568.2011.07887.x PMC3286125

[26] Mesgarani N, Chang EF (2012) Selective cortical representation of attended speaker in multi-talker speech perception. Nature 485: 233–236. 22522927 10.1038/nature11020 PMC3870007

[27] Zilany MSA, Bruce IC, Nelson PC, Carney LH (2009) A phenomenological model of the synapse between the inner hair cell and auditory nerve: long-term adaptation with power-law dynamics. J Acoust Soc Am 126: 2390–2412. 19894822 10.1121/1.3238250 PMC2787068

[28] McDermott J, Simoncelli E (2011) Sound texture perception via statistics of the auditory periphery: evidence from sound synthesis. Neuron 71: 926–940. 21903084 10.1016/j.neuron.2011.06.032 PMC4143345

[29] Miller GA, Nicely PE (1955) An analysis of perceptual confusions among some English consonants. J Acoust Soc Am 27: 338–352.

[30] Wang MD, Bilger RC (1973) Consonant confusions in noise: a study of perceptual features. J Acoust Soc Am 54: 1248–1266. 4765809 10.1121/1.1914417

[31] Phatak SA, Lovitt A, Allen JB (2008) Consonant confusions in white noise. J Acoust Soc Am 124: 1220–1233. 18681609 10.1121/1.2913251

[32] Woolley SMN, Casseday JH (2005) Processing of modulated sounds in the zebra finch auditory midbrain: responses to noise, frequency sweeps, and sinusoidal amplitude modulations. J Neurophysiol 94: 1143–1157. 15817647 10.1152/jn.01064.2004

[33] Louage DHG, van der Heijden M, Joris PX (2005) Enhanced temporal response properties of anteroventral cochlear nucleus neurons to broadband noise. J Neurosci 25: 1560–1570. 15703410 10.1523/JNEUROSCI.4742-04.2005 PMC6725990

[34] Attias H, Schreiner C (1997) Temporal low-order statistics of natural sounds. In: Advances in neural information processing systems, Cambridge, MA: MIT Press, volume 9. pp. 27–33.

[35] Singh NC, Theunissen FE (2003) Modulation spectra of natural sounds and ethological theories of auditory processing. J Acoust Soc Am 114: 3394–3411. 14714819 10.1121/1.1624067

[36] Wen B, Wang GI, Dean I, Delgutte B (2009) Dynamic range adaptation to sound level statistics in the auditory nerve. J Neurosci 29: 13797–13808. 19889991 10.1523/JNEUROSCI.5610-08.2009 PMC2774902

[37] deCharms RC, Blake DT, Merzenich MM (1998) Optimizing sound features for cortical neurons. Science 280: 1439–1444. 9603734 10.1126/science.280.5368.1439

[38] Linden JF, Liu RC, Sahani M, Schreiner CE, Merzenich MM (2003) Spectrotemporal structure of receptive fields in areas AI and AAF of mouse auditory cortex. J Neurophysiol 90: 2660–2675. 12815016 10.1152/jn.00751.2002

[39] Ahrens M, Linden J, Sahani M (2008) Nonlinearities and contextual inuences in auditory cortical responses modeled with multilinear spectrotemporal methods. J Neurosci 28: 1929–1942. 18287509 10.1523/JNEUROSCI.3377-07.2008 PMC6671443

[40] Chichilnisky EJ (2001) A simple white noise analysis of neuronal light responses. Network 12: 199–213. 11405422

[41] Simoncelli EP, Paninski L, Pillow J, Schwartz O (2004) Characterization of neural responses with stochastic stimuli. In: Gazzaniga M, editor, The cognitive neurosciences III, Cambridge, MA: MIT Press. pp. 327–338.

[42] Bialek W, Rieke F, Steveninck RdRv, Warland D (1991) Reading a neural code. Science 252: 1854–1857. 2063199 10.1126/science.2063199

[43] Mesgarani N, David SV, Fritz JB, Shamma SA (2009) Inuence of context and behavior on stimulus reconstruction from neural activity in primary auditory cortex. J Neurophysiol 102: 3329–3339. 19759321 10.1152/jn.91128.2008 PMC2804432

[44] Ramirez AD, Ahmadian Y, Schumacher J, Schneider D, Woolley SMN, et al (2011) Incorporating naturalistic correlation structure improves spectrogram reconstruction from neuronal activity in the songbird auditory midbrain. J Neurosci 31: 3828–3842. 21389238 10.1523/JNEUROSCI.3256-10.2011 PMC3273872

[45] Pasley BN, David SV, Mesgarani N, Flinker A, Shamma SA, et al (2012) Reconstructing speech from human auditory cortex. PLoS Biol 10: e1001251 doi:10.1371/journal.pbio.1001251 22303281 10.1371/journal.pbio.1001251 PMC3269422

[46] Dau T, Kollmeier B, Kohlrausch A (1997) Modeling auditory processing of amplitude modulation. i. detection and masking with narrow-band carriers. J Acoust Soc Am 102: 2892. 9373976 10.1121/1.420344

[47] Moore RC, Lee T, Theunissen FE (2013) noise-invariant neurons in the avian auditory cortex: hearing the song in noise. PLoS Comput Biol 9: e1002942 doi:10.1371/journal.pcbi.1002942 23505354 10.1371/journal.pcbi.1002942 PMC3591274

[48] Chechik G, Nelken I (2012) Auditory abstraction from spectro-temporal features to coding auditory entities. Proc Natl Acad Sci 109 (46) 18968–18973. 23112145 10.1073/pnas.1111242109 PMC3503225

[49] Schneider D, Woolley S (2013) Sparse and background-invariant coding of vocalizations in auditory scenes. Neuron 79: 141–152. 23849201 10.1016/j.neuron.2013.04.038 PMC3713513

[50] Woolley SMN, Fremouw TE, Hsu A, Theunissen FE (2005) Tuning for spectro-temporal modulations as a mechanism for auditory discrimination of natural sounds. Nat Neurosci 8: 1371–1379. 16136039 10.1038/nn1536

[51] Zion Golumbic EM, Ding N, Bickel S, Lakatos P, Schevon CA, et al (2013) Mechanisms underlying selective neuronal tracking of attended speech at a cocktail party. Neuron 77: 980–991. 23473326 10.1016/j.neuron.2012.12.037 PMC3891478

[52] Rees A, Green G, Kay R (1986) Steady-state evoked responses to sinusoidally amplitude-modulated sounds recorded in man. Hear Res 23: 123–133. 3745015 10.1016/0378-5955(86)90009-2

[53] Cunningham J, Nicol T, Zecker SG, Bradlow A, Kraus N (2001) Neurobiologic responses to speech in noise in children with learning problems: deficits and strategies for improvement. Clin Neurophysiol 112: 758–767. 11336890 10.1016/s1388-2457(01)00465-5

[54] Billings CJ, Tremblay KL, Stecker GC, Tolin WM (2009) Human evoked cortical activity to signal-to-noise ratio and absolute signal level. Hear Res 254: 15–24. 19364526 10.1016/j.heares.2009.04.002 PMC2732364

[55] Dahmen JC, Keating P, Nodal FR, Schulz A, King AJ (2010) Adaptation to stimulus statistics in the perception and neural representation of auditory space. Neuron 66: 937–948. 20620878 10.1016/j.neuron.2010.05.018 PMC2938477

[56] Cooper NP, Guinan JJ (2006) Efferent-mediated control of basilar membrane motion. J of Physiol 576: 4954. 10.1113/jphysiol.2006.114991 PMC1995651 16901947

[57] Hienz RD, Stiles P, May BJ (1998) Effects of bilateral olivocochlear lesions on vowel formant discrimination in cats. Hear Res 116: 10–20. 9508024 10.1016/s0378-5955(97)00197-4

[58] Guinan JJ (2006) Olivocochlear efferents: anatomy, physiology, function, and the measurement of efferent effects in humans. Ear Hear 27: 589–607. 17086072 10.1097/01.aud.0000240507.83072.e7

[59] Zilany MSA, Carney LH (2010) Power-law dynamics in an auditory-nerve model can account for neural adaptation to sound-level statistics. J Neurosci 30: 10380–10390. 20685981 10.1523/JNEUROSCI.0647-10.2010 PMC2935089

[60] Joris P, Yin T (1992) Responses to amplitude-modulated tones in the auditory nerve of the cat. J Acoust Soc Am 91: 215–232. 1737873 10.1121/1.402757

[61] Panzeri S, Senatore R, Montemurro MA, Petersen RS (2007) Correcting for the sampling bias problem in spike train information measures. J Neurophysiol 98: 1064–1072. 17615128 10.1152/jn.00559.2007

[62] Rothschild G, Nelken I, Mizrahi A (2010) Functional organization and population dynamics in the mouse primary auditory cortex. Nat Neurosci 13: 353–360. 20118927 10.1038/nn.2484

[63] Sakata S, Harris KD (2009) Laminar structure of spontaneous and sensory-evoked population activity in auditory cortex. Neuron 64: 404–418. 19914188 10.1016/j.neuron.2009.09.020 PMC2778614

[64] Averbeck BB, Latham PE, Pouget A (2006) Neural correlations, population coding and computation. Nat Rev Neurosci 7: 358–366. 16760916 10.1038/nrn1888

[65] Graf ABA, Kohn A, Jazayeri M, Movshon JA (2011) Decoding the activity of neuronal populations in macaque primary visual cortex. Nat Neurosci 14: 239–245. 21217762 10.1038/nn.2733 PMC3081541

[66] Jeanne J, Sharpee T, Gentner T (2013) Associative learning enhances population coding by inverting interneuronal correlation patterns. Neuron 78: 352–363. 23622067 10.1016/j.neuron.2013.02.023 PMC3641681

[67] Adibi M, McDonald JS, Clifford CWG, Arabzadeh E (2013) Adaptation improves neural coding efficiency despite increasing correlations in variability. J Neurosci 33: 2108–2120. 23365247 10.1523/JNEUROSCI.3449-12.2013 PMC6619115

[68] Slaney M (1993) An efficient implementation of the Patterson-Holdsworth auditory filter bank. Apple Computer, Perception Group, Tech Rep.

[69] Goodman D, Brette R (2008) Brian: a simulator for spiking neural networks in Python. Front Neuroinform 2: 5. 19115011 10.3389/neuro.11.005.2008 PMC2605403

[70] Glasberg BR, Moore BCJ (1990) Derivation of auditory filter shapes from notched-noise data. Hear Res 47: 103138. 10.1016/0378-5955(90)90170-t 2228789

[71] Ruggero MA (1992) Responses to sound of the basilar membrane of the mammalian cochlea. Curr Opin Neurobiol 2: 449–456. 1525542 10.1016/0959-4388(92)90179-o PMC3579517

[72] Dean I, Robinson BL, Harper NS, McAlpine D (2008) Rapid neural adaptation to sound level statistics. J Neurosci 28: 6430–6438. 18562614 10.1523/JNEUROSCI.0470-08.2008 PMC6670892

