# Supplementary figures and images for: Constructing Noise-Invariant Representations of Sound in the Auditory Pathway
Source: PLoS Biol. 2013 Nov 12;11(11):e1001710. doi: 10.1371/journal.pbio.1001710 (PMC3825667; doi:10.1371/journal.pbio.1001710)

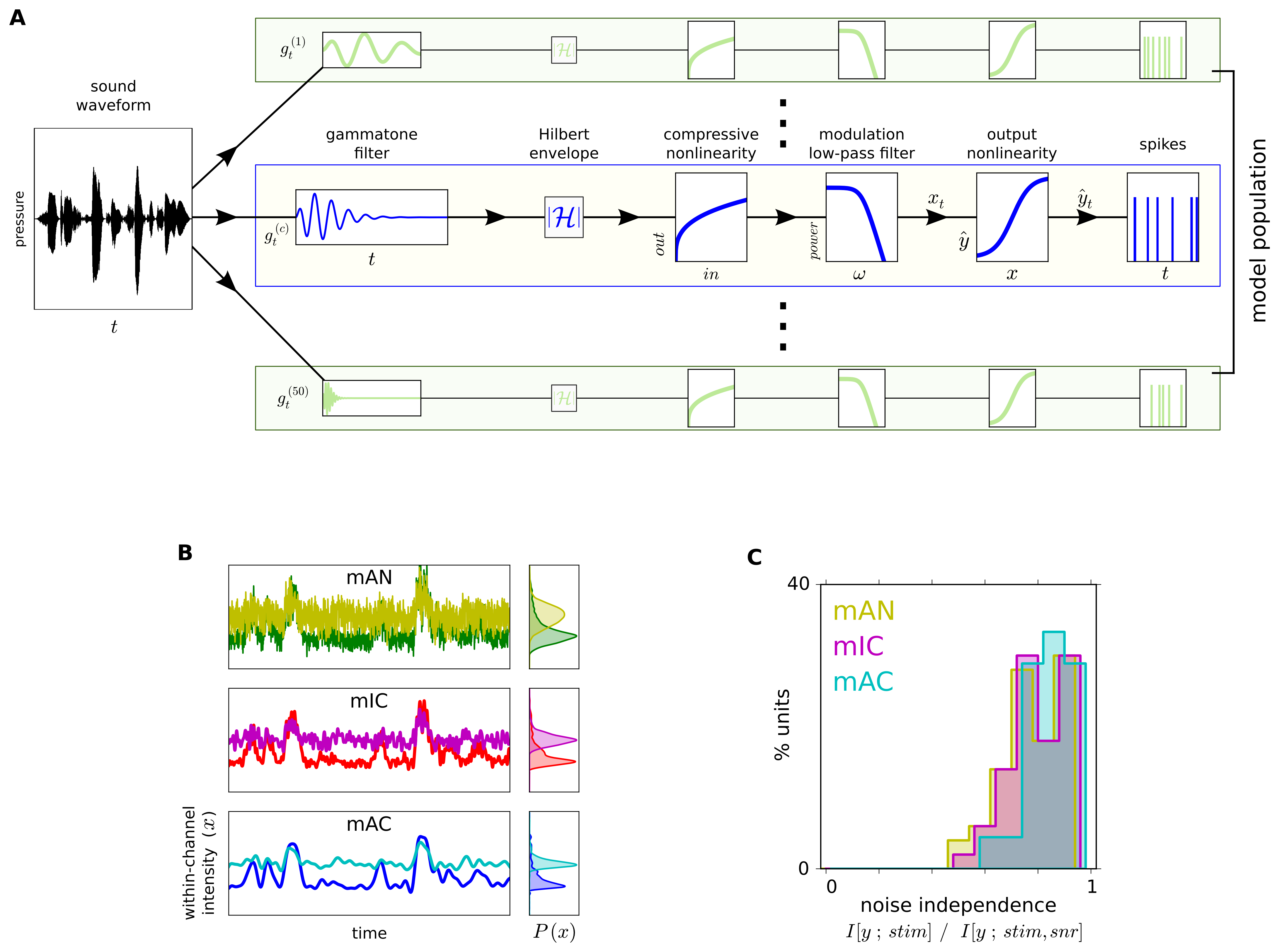

Supplement: Figure S1 — Increasing independence of response distributions to background noise level cannot be explained by increased modulation filtering. This figure shows a simulated experiment designed to test whether the results in Figure 2 could be explained by changes in the temporal integration properties of neurons in the auditory pathway. We constructed populations of model auditory neurons, simulated their responses to the natural sounds presented in the main text, and performed the same analysis as in Figure 2. The populations were identical except for the parameter , defining the temporal integration properties of the model neurons. Further details follow, but in brief, (A) shows a general schematic for how the model neurons process sound stimuli, (B) illustrates how affects input signals in the model, and (C) is a direct analogue of Figure 2C, using the model neurons. (A) Model of auditory neurons used in the simulation. This comprises two stages. The first stage is a simple model of cochlear filtering. We began with the pressure waveforms of the natural sounds used in the main text. We simulated frequency-selective cochlear channels by filtering the sound waveforms through a gammatone filterbank. This was implemented as a set of 50 IIR gammatone filters [68], using the Brian simulator [69] in Python. Filter CFs were ERB-spaced between 250 Hz and 20 kHz, as in ref. [70]. We next extracted the amplitude envelope of each filter output, via the magnitude of the Hilbert transform. We then applied a compressive nonlinearity to envelopes to approximate the amplitude compression that occurs at the cochlea [71]. In the second stage, we constructed populations of model auditory neurons, based on the output of the 50 cochlear channels. Populations were defined by the choice of a single parameter, , which characterizes the temporal integration properties of the model neurons in each population. We assumed that each auditory neuron within a model population received input from only one pe [file pbio.1001710.s001.tif]

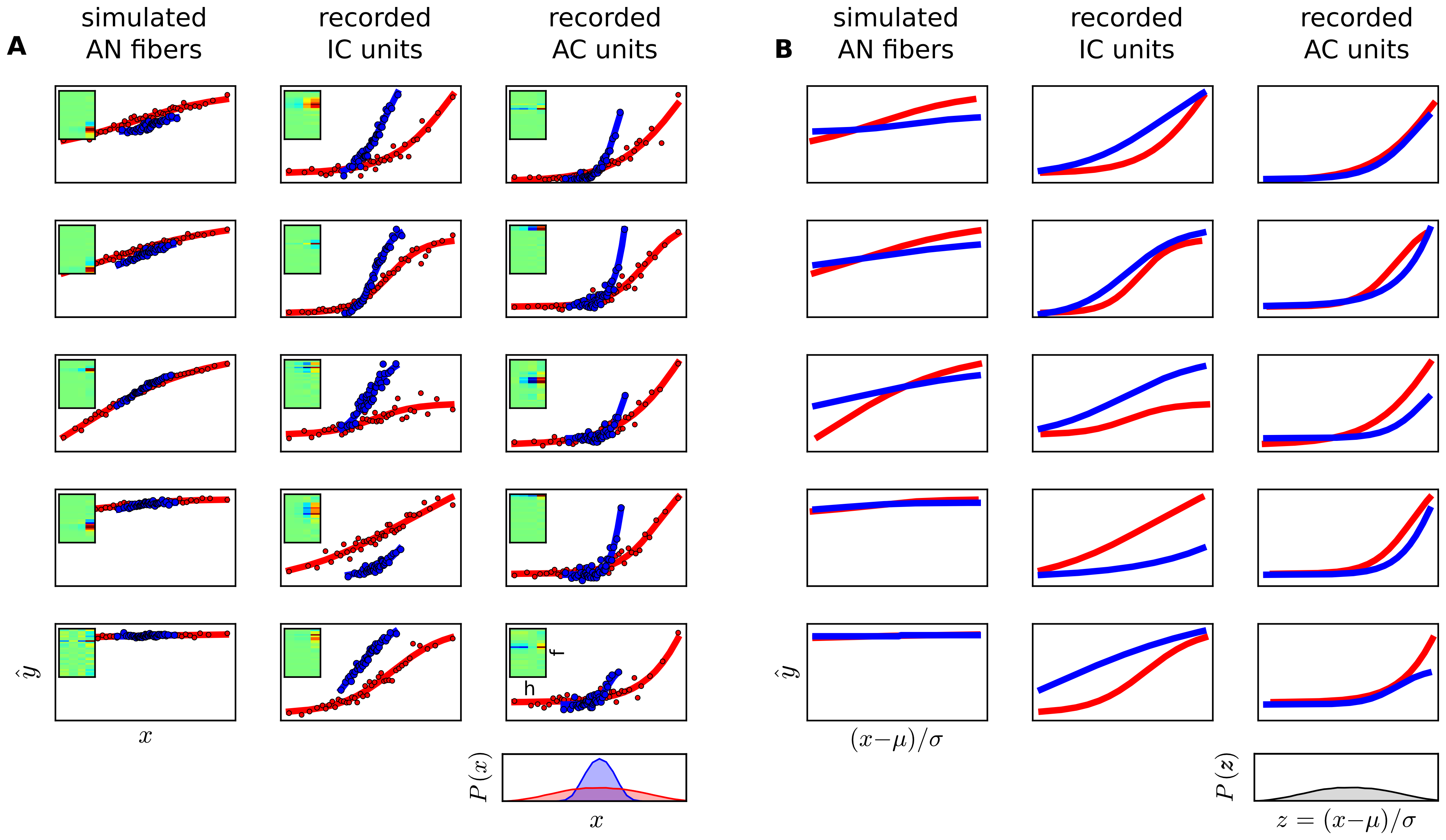

Supplement: Figure S2 — Further examples of adaptation to contrast, as shown in Figure 5B–C . In addition to the general trend of an increasing slope of the nonlinearity with contrast, some sAN fibers (Examples 1 and 2) underwent small shifts in mean level at lower contrast; greater effects were seen in some IC units (Examples 4 and 5). Some IC units showed other contrast-dependent changes to nonlinearities, including horizontal shifts (Example 1) and changes in saturation points (Example 3). While more complex models of contrast-dependent changes to nonlinearities were sometimes needed to characterize the behavior of IC neurons (such as the more general classes of contrast kernel models described in ref. [12]),changes in slope for IC units were, overall, smaller than in cortex, but larger than in the sAN. (TIFF) [file pbio.1001710.s002.tif]

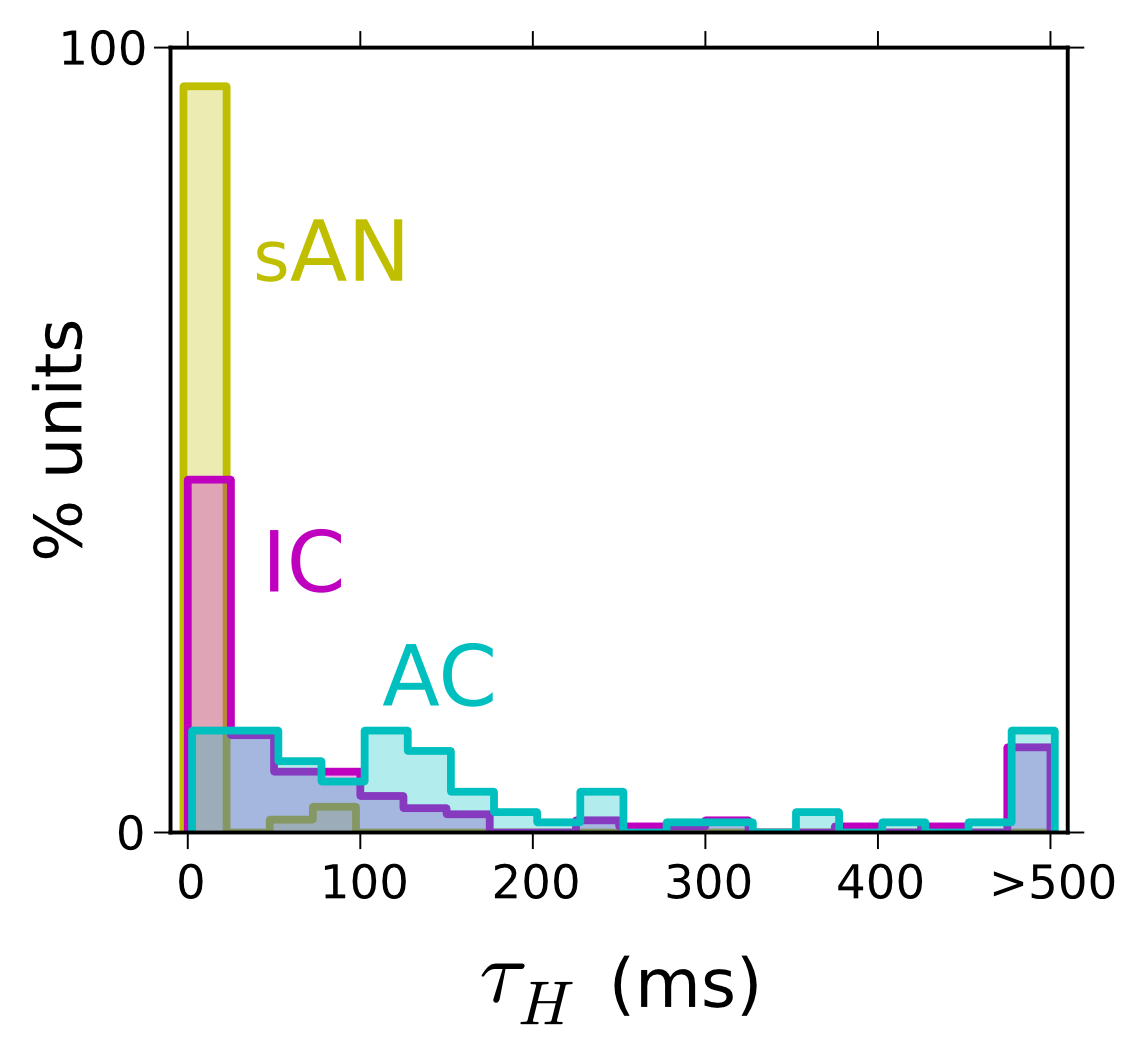

Supplement: Figure S3 — Fitted time constants for gain control at different levels of the auditory pathway. These time constants were obtained using the same stimuli and procedure as previously documented [12]. After a change in the spectral pattern of contrast of a DRC, the gain of IC and cortical units' nonlinearities changed with an approximately exponential time course, with median time constants of 35 ms in IC and 117 ms in AC. Contrast-dependent gain changes were generally weak or nonexistent in the sAN, with estimated time constants being below 25 ms (and hence not detectable with this method). Pairwise differences significant at (rank-sum tests). (TIFF) [file pbio.1001710.s003.tif]

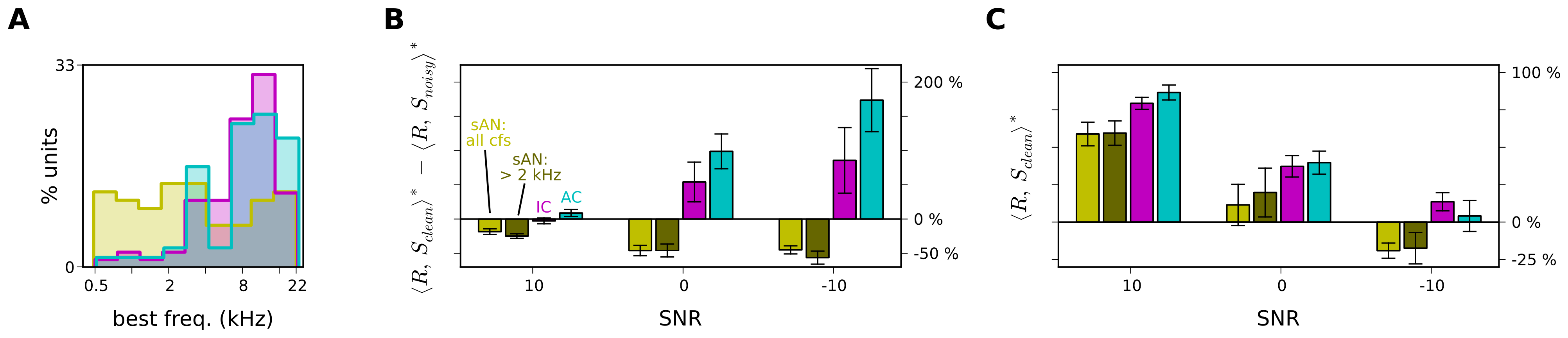

Supplement: Figure S4 — The more uniform coverage of frequency space by the simulated AN population does not explain the decoding results in the main text. (A) Histogram of best frequencies of units in each location. (B, C) The more uniform frequency coverage by the population of sAN fibers, compared with that of the measured IC and cortical populations, could not explain the differences in normalized decoder performance shown in Figure 7D–E. Here, we halved the sAN population in size, keeping only the simulated fibers with higher CFs (>2 kHz). This produced near identical values of (B) and (C) to the full sAN population. While these relative metrics remained unaffected, the absolute performance of the decoder for the clean sound () was lower for the high-CF subpopulation than the full sAN population (not shown). This is consistent with the trends shown in Figure 7A: since the high-CF subpopulation contained only 42 simulated fibers (rather than the full 85), there was less information available for inference. However, for the high-CF subpopulation was lower than that predicted by Figure 7A: subpopulations of 42 randomly selected fibers (i.e., with more uniform coverage of the spectrum) yielded values of that were on average 10 percentage points higher than the high-CF subpopulation. Thus we can conclude that the greater coverage of the frequency spectrogram by the population of simulated AN fibers, compared with that of the measured IC and cortical populations, contributes to the better absolute decoder performance for the clean sound () in the sAN. (TIFF) [file pbio.1001710.s004.tif]

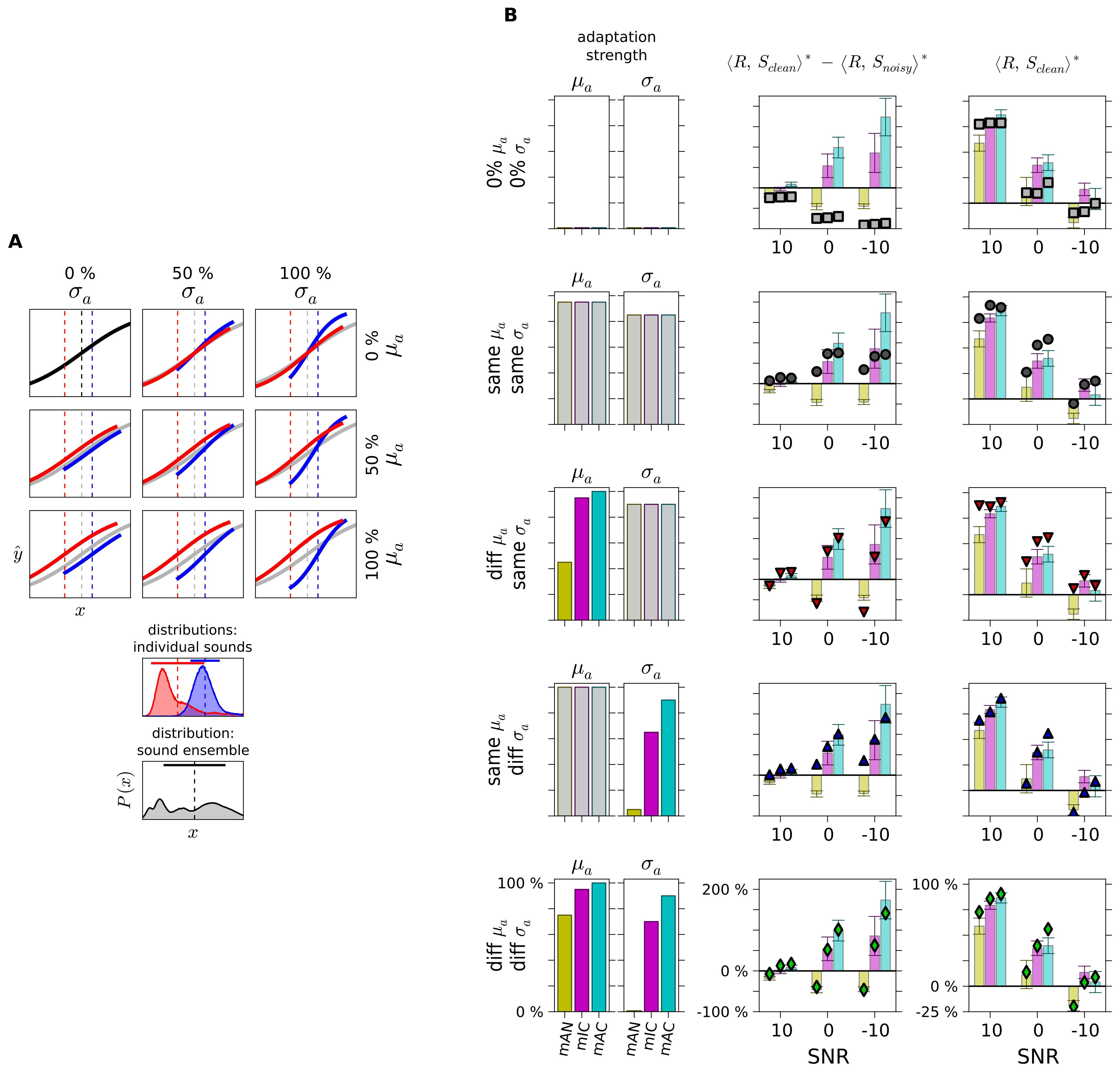

Supplement: Figure S5 — Simulation of how both temporal integration and adaptation affect the population encoding of complex sounds, with and without background noise. This figure shows simulated experiments designed to test whether the results in Figure 7D and 7E could be explained by changes in the temporal integration and/or adaptation properties of neurons in the auditory pathway. As in Figure S1, we constructed populations of model auditory neurons, simulated their responses to the natural sounds presented in the main text, and performed the same decoding analyses as in the main text. The simulation was similar to that performed in Figure S1, and thus followed the same schema as in Figure S1A. However, Figure S1 only considered populations of neurons that differed in their temporal integration properties. Here, we simulated populations that also differed in the strength of their adaptation to stimulus statistics. We constructed populations of model neurons that were identical to each other, except for the value of three parameters: , defining the temporal integration properties of the model neurons (as in the simulations in Figure S1); , defining the strength of the model neurons' adaptation to the mean intensity; and , defining the strength of the model neurons' adaptation to the stimulus contrast. Varying these parameters allowed us to test hypotheses about the factors underlying the results in Figure 7D–E. For each population, the values of and affected the operation of each neuron's sigmoidal output nonlinearity. The shapes of these output nonlinearities were allowed to vary as a function of stimulus statistics, in order to impart adaptation to the neuron. Thus, for each model location (defined by ), and each set of adaptation parameters ( and ), we generated a set of spike data of the same form as that used in the main text. Further details follow, but in brief: (A) illustrates how and affect the output nonlinearities of neurons in the model; (B) shows the results of fitting thes [file pbio.1001710.s005.tif]

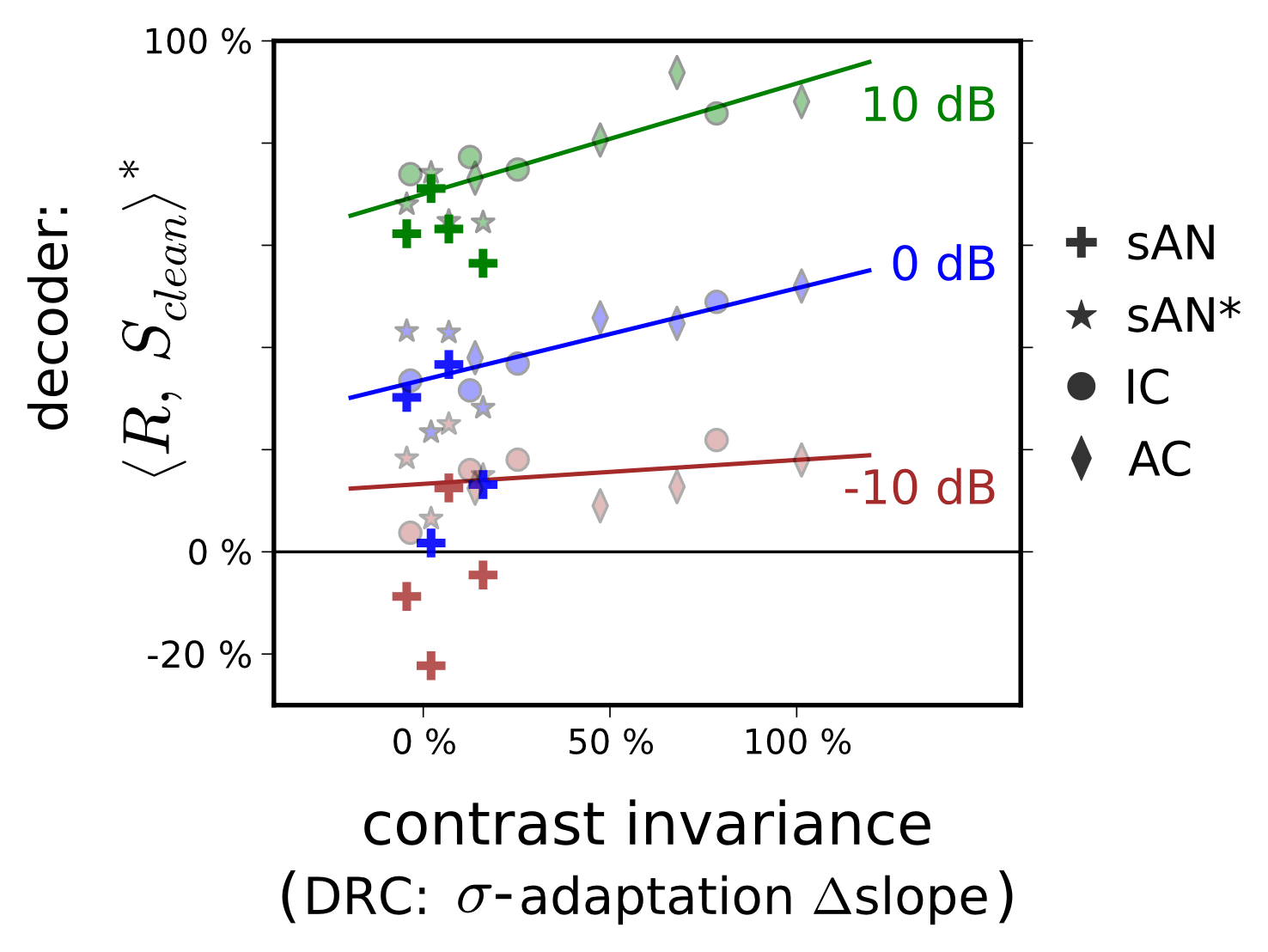

Supplement: Figure S6 — Adjusted for sAN units in Figure 8B . The results of Figure 8B show the relationship between the strength of σ-adaptation and the noise-tolerance of encoding. However, is also affected by BI (Figure 8A). Because the sAN units had low BI (Figure 4B), decoding the responses of the sAN population to noisy sounds produced spectrograms that included the noise present in but not (Figure 6); as a result, was even lower for the sAN. Therefore, to elucidate the relationship between -adaptation and the noise-tolerance of encoding, we compensated for the low BI of sAN units in that figure. As described in Materials and Methods, this involved using a baseline-corrected similarity metric, which ignored the difference in mean between the decoded and clean spectrograms. Here, we show the effect of that compensation on . Pluses show the uncorrected metric for the sAN; stars show the corrected metrics as in Figure 8B. The correction had little to no impact on for IC and cortical subpopulations; for the IC and AC data points on this plot, the difference between corrected and uncorrected metrics differed by an average of 0.5% (and hence are not depicted). (TIFF) [file pbio.1001710.s006.tif]

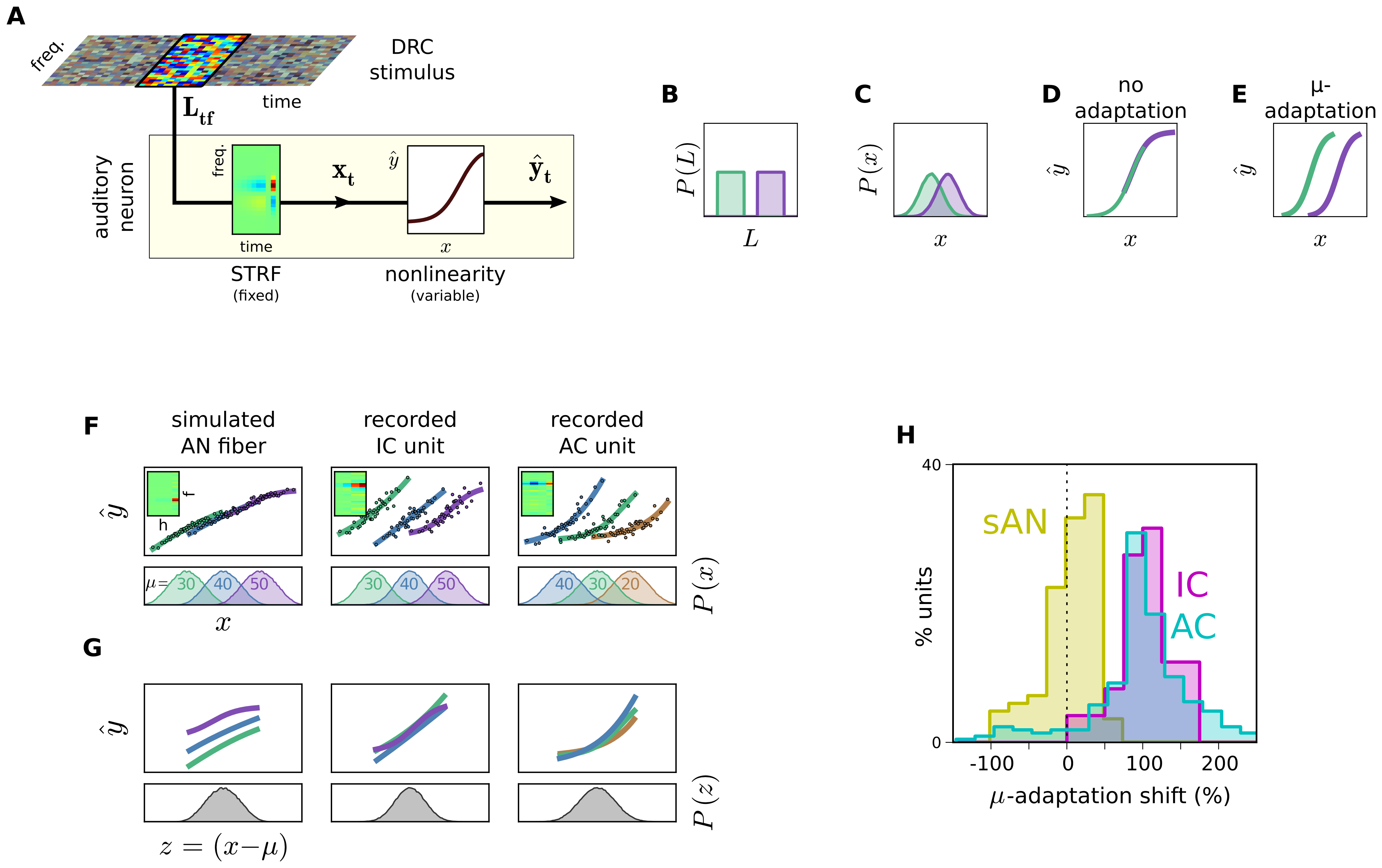

Supplement: Figure S7 — A separate set of experiments characterizing adaptation to the mean stimulus intensity in sAN, IC, and AC neurons. (A) Schematic of a LN model. In this experiment, we probed auditory neurons using DRC stimuli. As in the experiment presented in Figure 5, these were constructed as superpositions of tones, whose time-varying levels, , were drawn from particular distributions (shown in B). The transformation of the sound into a time-varying spike rate () is modeled as a two-stage procedure: first, the sound spectrogram (; top and bottom; colors denote tone level) is filtered through a linear STRF. This reduces the large dimensionality of the input space to a 1D time-varying signal, . Second, this signal is passed through a sigmoidal output nonlinearity, yielding the firing rate (). (B) Statistics of the DRCs were controlled by varying the distribution of tone levels, . In this set of experiments, the mean () of was varied (cf., the experiment shown in Figure 5, where the width of was varied). (C) For each unit, the distribution of STRF-filtered DRCs, , depends on the distributions shown in (B). (D) Illustration of a fixed output nonlinearity for an idealized neuron with no adaptation to the mean. The two colors show the portion of the nonlinearity that would be explored by the stimulus distributions shown in (B) and (C). (E) Illustration of two output nonlinearities for an idealized neuron with complete (dynamic-range) adaptation to the mean. This neuron no longer has a single fixed output nonlinearity; rather, the nonlinearity is horizontally shifted to cover the presented range of values. (F) Data from example units in each location. These show how output nonlinearities change as the mean tone level () changed. STRFs (insets) range from 0.5 kHz to 22.6 kHz on the frequency () axis, and are shown over only 100 ms of the 200 ms history () at 25 ms resolution. Colors denote nonlinearities in different mean-level conditions; corresponding distributions shown below. For th [file pbio.1001710.s007.tif]

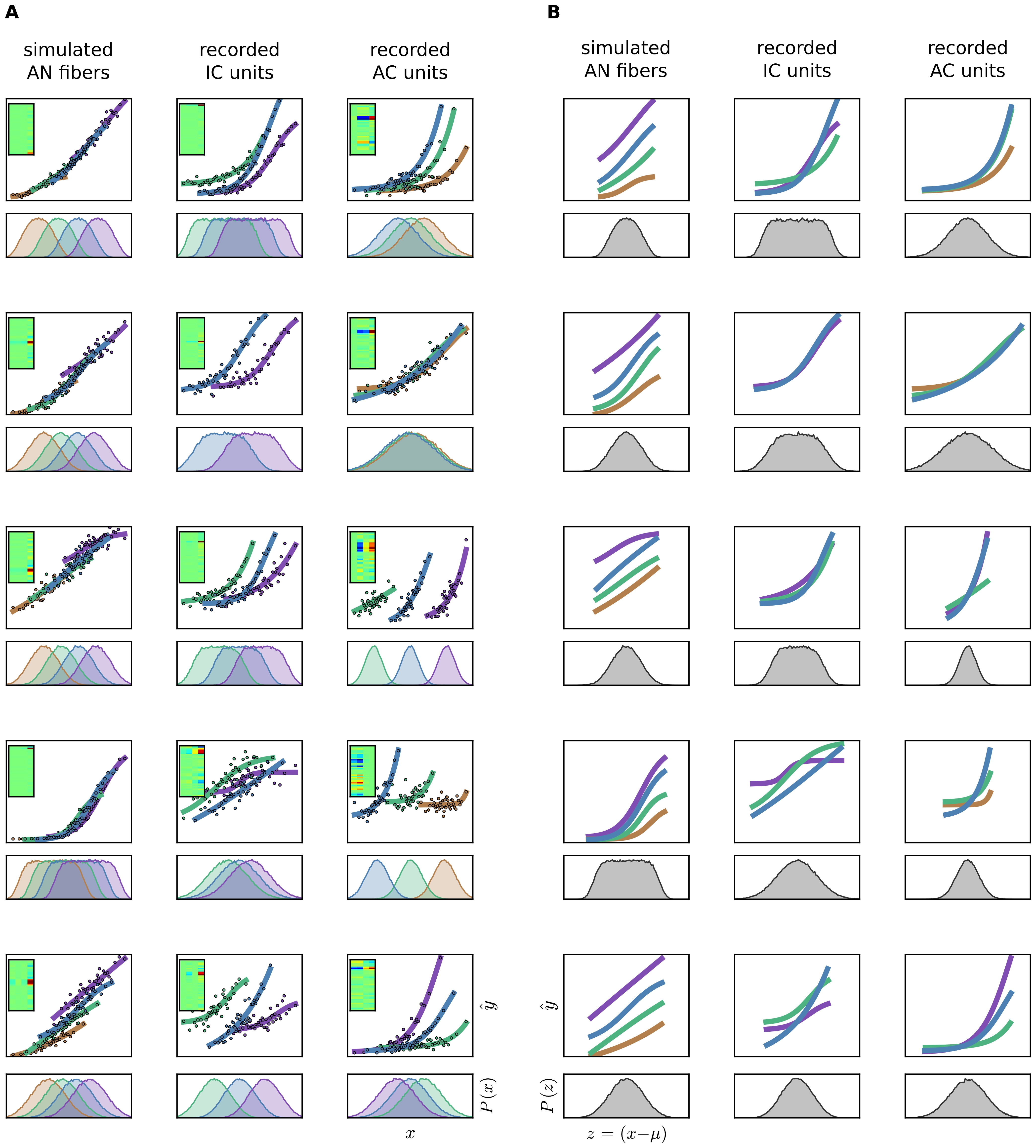

Supplement: Figure S8 — Further examples of adaptation to mean tone level, as shown in Figure S7F–G. (A) Output nonlinearities for five example sAN fibers (left), five IC units (middle), and five cortical units (right). Insets show units' STRFs, as in Figure 5B. For each example, top panel shows the fitted output nonlinearities for DRCs presented at different mean levels. All DRCs were constructed of pure tones; tones had levels drawn from a uniform distribution with halfwidth dB, and means of dB SPL (orange), dB SPL (green), dB SPL (blue), or dB SPL (purple). Three to four of these conditions were usually presented for each unit; some IC units were only tested with two conditions. Using the LN model shown in Figure S7A, the DRC stimuli produced from each of these tone-level distributions are filtered through units' STRFs to produce time-varying signals, . The statistics of for each condition are a function of the coefficients in the STRF. Thus, the distributions vary from unit to unit in a number of ways. For example, STRFs dominated by a single coefficient (e.g., sAN Example 4, IC Example 1) have more uniform-like , while STRFs with a large number of nonzero coefficients are more Gaussian-like (e.g., most cortical units). Also, the net balance between excitatory (red) and inhibitory (blue) coefficients of the STRF determine how increasing μ changes the mean of the distribution . With more excitation in the STRF (most examples), increased for larger; with more inhibition, decreased for larger (AC Examples 1, 4, and 5). In a small number of cases, excitation and inhibition were approximately equal (AC Example 2), such that did not change considerably with . (B) Output nonlinearities for the units in (A), replotted as a function of normalized coefficients, , as in Figure S7G. As in Figure 5B–C, output nonlinearities were generally independent of in the sAN, but changed considerably with mean level in the IC and cortex. The trend was such that in these higher stages of the pathway, responses [file pbio.1001710.s008.tif]

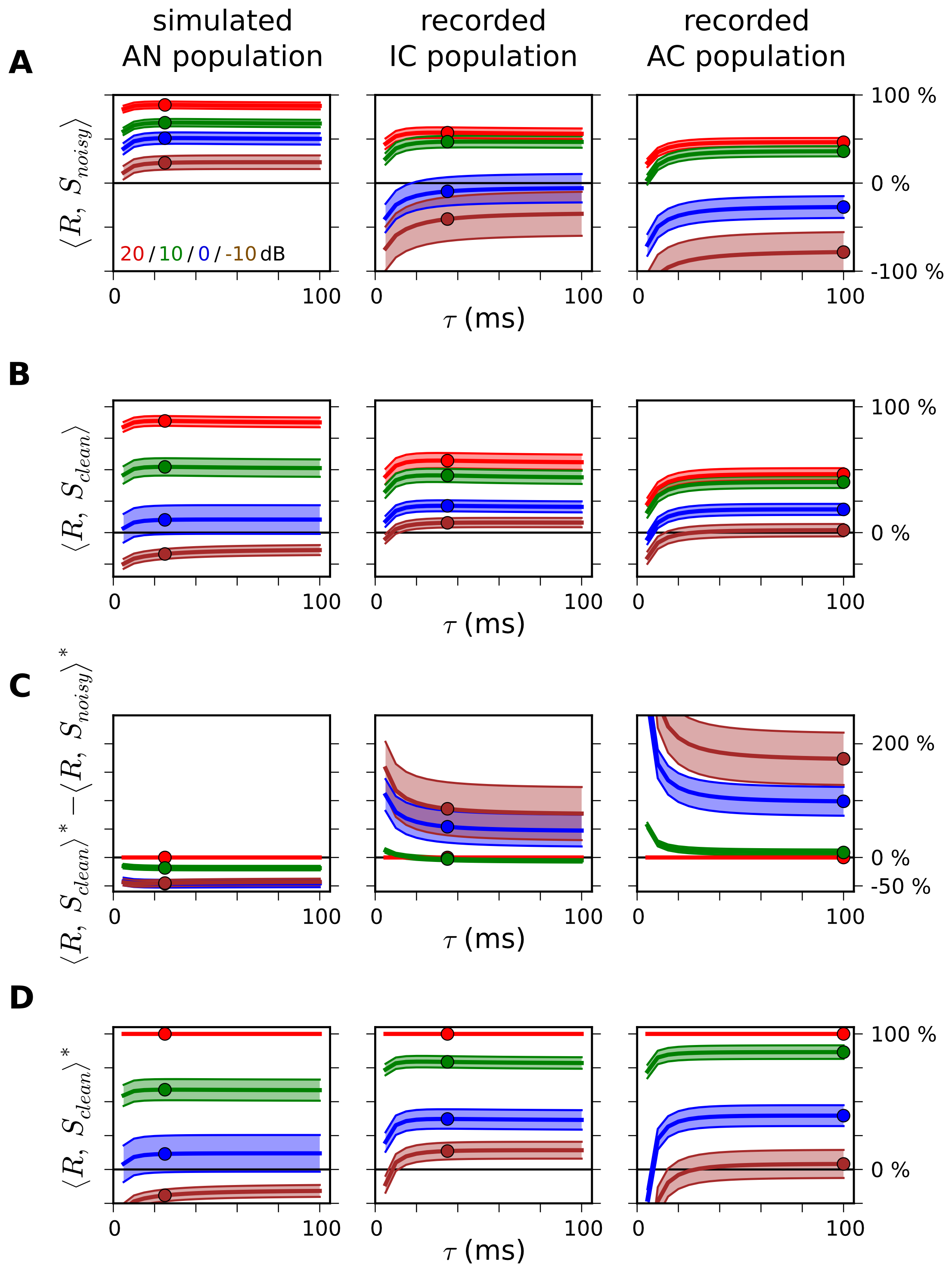

Supplement: Figure S9 — Differences in decoder performance were not the result of the time constants used to reconstruct spectrograms. As described in Materials and Methods, the decoder constructs an estimate of the recent spectrogram history for each 5 ms bin. In order to integrate these successive estimates into a single decoded spectrogram, we convolved the set of estimates with exponential kernels, , where ms for sAN, 35 ms for IC, and 100 ms for AC. Here, similarity metrics as used in the main text are shown for values of τ ranging from 5 ms to 100 ms. As in Figure 7, shaded regions show 95% confidence intervals. Filled circles show the τ values used in the main text; these were chosen to maximize for each location. However, values of between 25 ms and 100 ms produced very similar results for all locations. (TIFF) [file pbio.1001710.s009.tif]

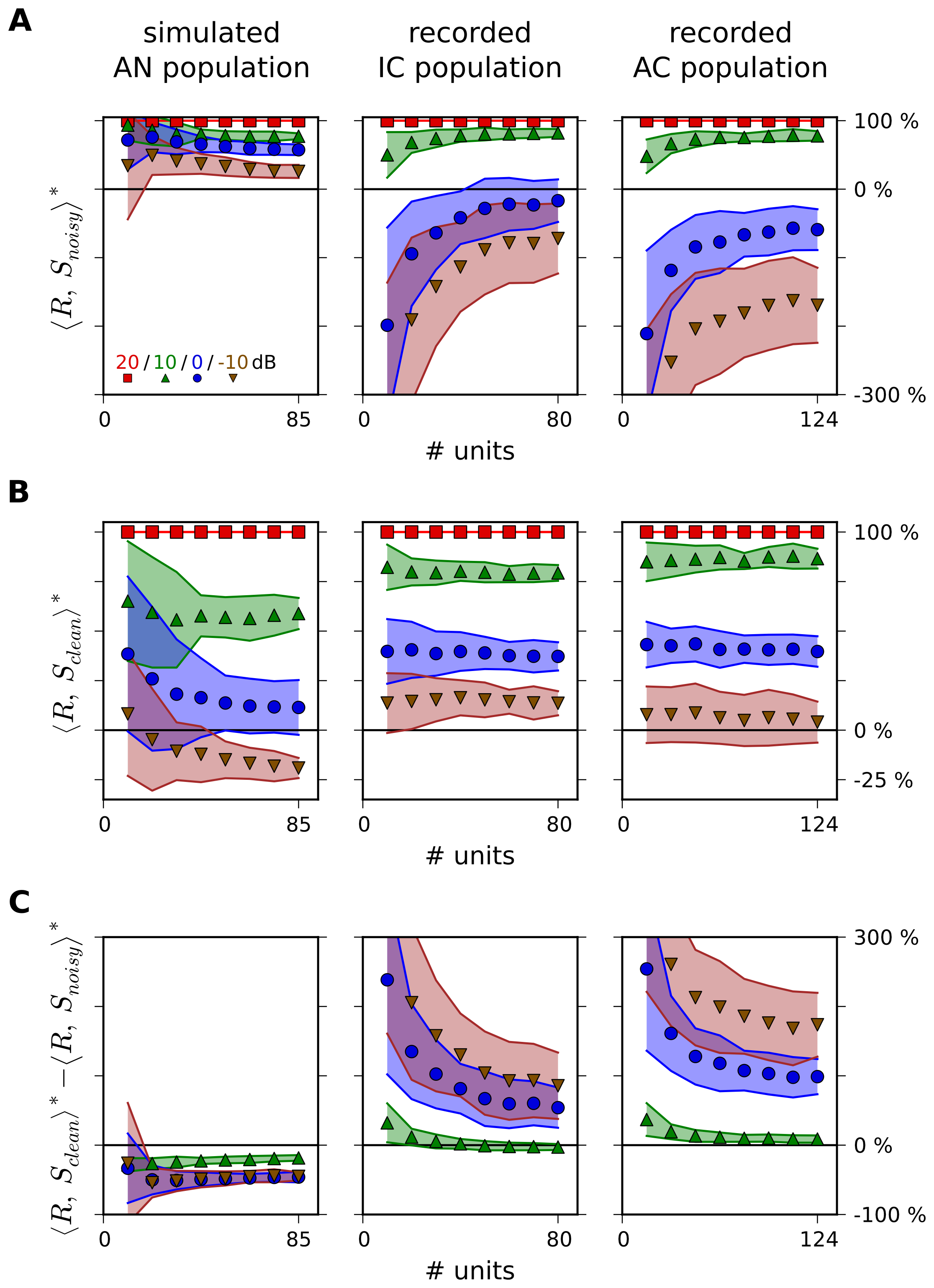

Supplement: Figure S10 — Stability of metrics with increasing population size. In Figure 7A, we show that the values of the decoder metric generally increased as more units were included in the analysis. Here, we show how the normalized metrics (A) , (B) , and (C) converged to stable values as the number of units included in the analysis was increased. Thus, the differences across location in the normalized decoder metrics shown in Figure 7D–E are not the result of differences in the absolute fidelity of the decoding. (TIFF) [file pbio.1001710.s010.tif]
