# Supplementary material for: Constructing Noise-Invariant Representations of Sound in the Auditory Pathway
Source: PLoS Biol. 2013 Nov 12;11(11):e1001710. doi: 10.1371/journal.pbio.1001710 (PMC3825667; doi:10.1371/journal.pbio.1001710)
Supplement: Table S1 — Contributions of increasing BI and CI along the auditory pathway to the results in Figure 8 . In Figure 8A, we demonstrate that the shift from -representations in the sAN population to -representations in the AC population can largely be explained by an increase in neurons' BI along the auditory pathway. In Figure 8B, we demonstrate that the increasing robustness of encoding can largely be explained by an increase in neurons' contrast invariance along the auditory pathway. This table documents the statistics for these two figures (A for Figure 8A; B for Figure 8B). The percentages shown quantify the contributions of BI and CI toward explaining the differences between the decoder metrics across locations. The values are relative effect sizes within a general linear model. They were calculated by fitting a set of multiple linear regression models (ANCOVA) to (A) the data points in Figure 8A (where the decoder metric is ) and (B) Figure 8B (where the decoder metric is ). The first row of the table considers only the differences between sAN and IC data (for each of A and B, n = 24 data points = 3 SNRs × 4 subpopulations × 2 locations); the second row considers only the differences between IC and AC data (24 data points); while the third row considers the differences across all three locations (36 data points). To calculate relative effect sizes for (A), we fitted the following four linear models:(14) (15) (16) (17)where , , , and are categorical variables. Model is the reference model; model adds BI as an explanatory variable, adds CI, and captures across-location differences that remain unexplained by BI and CI. Denoting the residual variance for model as , the relative effect size of BI was calculated as . The relative effect size of CI was calculated as . The unexplained portion was calculated as . The procedure for calculating relative effect sizes for (B) was identical, except the order of adding BI and CI to the multiple linear regression model was reversed. (PDF) [file pbio.1001710.s011.pdf]

Supplementary Tables

Table S1

A $\langle R,\, S_{clean}\rangle^* - \langle R,\, S_{noisy}\rangle^*$

	BI contribution (μ-adaptation)	CI contribution (σ-adaptation)
AN → IC	98 %	0 %
IC → AC	92 %	6 %
AN → IC → AC	**98 %**	**1 %**

B $\langle R,\, S_{clean}\rangle^*$

	BI contribution (μ-adaptation)	CI contribution (σ-adaptation)
AN → IC	34 %	61 %
IC → AC	14 %	85 %
AN → IC → AC	**22 %**	**70 %**

Contributions of increasing baseline invariance (BI) and contrast invariance (CI) along the auditory pathway to the results in Fig. 8.

In Fig. 8A, we demonstrate that the shift from S_{noisy}-representations in the sAN population, to S_{clean}-representations in the AC population, can largely be explained by an increase in neurons' baseline invariance (BI) along the auditory pathway. In Fig. 8B, we demonstrate that the increasing robustness of S_{clean} encoding can largely be explained by an increase in neurons' contrast invariance along the auditory pathway.

This table documents the statistics for these two figures (**A** for Fig. 8A; **B** for Fig. 8B). The percentages shown quantify the contributions of BI and CI towards explaining the differences between the decoder metrics across locations.

The values are relative effect sizes within a general linear model. They were calculated by fitting a set of multiple linear regression models (ANCOVA) to (**A**) the data points in Fig. 8A (where the decoder metric is $Y = \langle R,\, S_{clean}\rangle^* - \langle R,\, S_{noisy}\rangle^*$), and (**B**) Fig. 8B (where the decoder metric is $Y = \langle R,\, S_{clean}\rangle^*$). The first row of the table considers only the differences between sAN and IC data (for each of **A** and **B**, n=24 data points = 3 SNRs × 4 subpopulations × 2 locations); the second row considers only the differences between IC and AC data (24 data points); while the third row considers the differences across all three locations (36 data points).

To calculate relative effect sizes for **A**, we fitted the following four linear models:

$$M_0 : \quad Y = a_{snr} \tag{1}$$
$$M_1 : \quad Y = a_{snr} + (b_{snr} \times BI) \tag{2}$$
$$M_2 : \quad Y = a_{snr} + (b_{snr} \times BI) + (c_{snr} \times CI) \tag{3}$$
$$M_3 : \quad Y = d_{snr,loc} + (b_{snr} \times BI) + (c_{snr} \times CI) \tag{4}$$

where $a_{snr}, b_{snr}, c_{snr}$ and $d_{snr,loc}$ are categorical variables. Model M_0 is the reference model; model M_1 adds BI as an explanatory variable; M_2 adds CI; and M_3 captures across-location differences that remain unexplained by BI and CI. Denoting the residual variance for model M_i as V_i, the relative effect size of BI was calculated as $(V_0 - V_1)/(V_0 - V_3)$. The relative effect size of CI was calculated as $(V_1 - V_2)/(V_0 - V_3)$. The unexplained portion was calculated as $(V_2 - V_3)/(V_0 - V_3)$.

The procedure for calculating relative effect sizes for **B** was identical, except the order of adding BI and CI to the multiple linear regression model was reversed.
